# Metabolite profiling of human‐originated Lachnospiraceae at the strain level

**DOI:** 10.1002/imt2.58

**Published:** 2022-10-13

**Authors:** Rashidin Abdugheni, Wen‐Zhao Wang, Yu‐Jing Wang, Meng‐Xuan Du, Feng‐Lan Liu, Nan Zhou, Cheng‐Ying Jiang, Chang‐Yu Wang, Linhuan Wu, Juncai Ma, Chang Liu, Shuang‐Jiang Liu

**Affiliations:** ^1^ State Key Laboratory of Microbial Resources, Environmental Microbiology Research Center (EMRC) Institute of Microbiology, Chinese Academy of Sciences Beijing China; ^2^ State Key Laboratory of Desert and Oasis Ecology Xinjiang Institute of Ecology and Geography, Chinese Academy of Sciences Urumqi China; ^3^ State Key Laboratory of Mycology Institute of Microbiology, Chinese Academy of Sciences Beijing China; ^4^ University of Chinese Academy of Sciences Beijing China; ^5^ State Key Laboratory of Microbial Technology Shandong University Qingdao China; ^6^ College of Life Sciences Hebei University Baoding China; ^7^ Colleg of Life Sciences University of Science and Technology of China Hefei China

**Keywords:** alcohols, aldehydes, Blautia, Lachnospiraceae, metabolite profiling, phenols, short‐chain fatty acids

## Abstract

The human gastrointestinal (GI) tract harbors diverse microbes, and the family Lachnospiraceae is one of the most abundant and widely occurring bacterial groups in the human GI tract. Beneficial and adverse effects of the Lachnospiraceae on host health were reported, but the diversities at species/strain levels as well as their metabolites of Lachnospiraceae have been, so far, not well documented. In the present study, we report on the collection of 77 *h*uman‐originated *L*a*ch*nospiraceae *sp*ecies (please refer hLchsp, https://hgmb.nmdc.cn/subject/lachnospiraceae) and the in vitro metabolite profiles of 110 Lachnospiraceae strains (https://hgmb.nmdc.cn/subject/lachnospiraceae/metabolites). The Lachnospiraceae strains in hLchsp produced 242 metabolites of 17 categories. The larger categories were alcohols (89), ketones (35), pyrazines (29), short (C2–C5), and long (C > 5) chain acids (31), phenols (14), aldehydes (14), and other 30 compounds. Among them, 22 metabolites were aromatic compounds. The well‐known beneficial gut microbial metabolite, butyric acid, was generally produced by many Lachnospiraceae strains, and *Agathobacter rectalis* strain Lach‐101 and *Coprococcus comes* strain NSJ‐173 were the top 2 butyric acid producers, as 331.5 and 310.9 mg/L of butyric acids were produced in vitro, respectively. Further analysis of the publicly available cohort‐based volatile‐metabolomic data sets of human feces revealed that over 30% of the prevailing volatile metabolites were covered by Lachnospiraceae metabolites identified in this study. This study provides Lachnospiraceae strain resources together with their metabolic profiles for future studies on host–microbe interactions and developments of novel probiotics or biotherapies.

## Highlights

- The human‐originated Lachnospiraceae biobank included 77 species was constructed.
- In vitro metabolite profiling of 110 Lachnospiraceae strains yielded 242 metabolites of 17 categories.
- Many Lachnospiraceae strains produce SCFAs, and *Agathobacter rectalis* strain Lach‐101 and *Coprococcus comes* strain NSJ‐173 are the top two butyric acid producers in vitro.

## Graphical abstract

The human‐originated Lachnospiraceae biobank included 77 species was constructed. In vitro metabolite profiling of 110 Lachnospiraceae strains yielded 242 metabolites of 17 categories. Many Lachnospiraceae strains produce short‐chain fatty acids, and *Agathobacter rectalis* strain Lach‐101 and *Coprococcus comes* strain NSJ‐173 are the top two butyric acid producers in vitro.

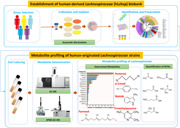

## INTRODUCTION

Members of the family Lachnospiraceae are prevalent and globally distributed in human guts [1, 2, 3, 4, 5]. All the members of Lachnospiraceae are strictly anaerobic, Gram stain positive or negative, and can ferment a variety of substrates, such as cellobiose and fructose, and produce a variety of metabolites, including short‐chain fatty acids (SCFAs) [6, 7, 8]. According to an integrated analysis of 75 different studies on human gut metagenomic data sets, the Lachnospiraceae taxa accounted for approximately 10% of the total gut microbiomes [9]. In addition, Lachnospiraceae was detected in subjects of different age groups, including infants [10, 11], teenagers [12, 13], young and middle‐aged adults [14, 15], and elderly people [16, 17, 18]. The high prevalence and abundance, and lifelong associations with human beings suggest that Lachnospiraceae possibly plays important roles in human health and diseases throughout their lives. Indeed, both beneficial and harmful effects of Lachnospiraceae on host health have been reported: The members of Lachnospiraceae, such as *Roseburia homins*, *Blautia producta*, and *Roseburia intestinalis*, and *Anaerobutyricum hallii* produce SCFAs and vitamins, and they were reported to have anti‐inflammatory, immunity‐inducing, and homeostasis‐maintaining effects [19]. As one of the most well‐studied SCFAs, butyric acid was reported to be a preferred energy source for colonocytes [20] and affects peripheral organs indirectly by activation of hormonal and nervous systems [21]. Intestinal microbiota produces many aromatic compounds, but only a few of them were well studied, among which equol was reported to reduce the risk of prostate cancer [22], 2,4‐di‐*tert*‐butylphenol was an antipathogenic compound [23], while some aromatic compounds, including *p*‐cresol and indole, were reported to be toxic to host health [24, 25]. For the beneficial effects, certain members of Lachnospiraceae were characterized as commercial probiotics, such as *R. homins*, which was patented for probiotics (the United States, Patent No. US9314489) [26]. Studies based on germ‐free mice revealed that Lachnospiraceae isolates suppressed *Clostridium difficile* infection [27]. Despite the beneficial effects, metagenomic studies showed that increased abundances of genera Blautia, Dorea, and Mediterraneibacter may contribute to host obesity [28, 29, 30, 31]. Increased abundances of Blautia species and *Mediterraneibacter gnavus* were observed in subjects with inflammatory bowel disease and primary sclerosing cholangitis [29, 32], although other studies reported contradictory results [33, 34]. Members of Anaerostipes, Blautia, Dorea, Roseburia, and Coprococcus were reportedly associated with the occurrences of major depressive disorder and Crohn's disease [35]. Further culture‐based cause‐and‐effect studies confirmed the functions of Lachnospiraceae members [36, 37, 38, 39]. For example, *Roseburia hominis* alleviated neuroinflammation via SCFA production, *Blautia wexlerae* ameliorated obesity and type 2 diabetes via gut microbiota remodeling [40, 41] and *Agathobacter rectalis* suppressed lymphomagenesis [42] and attenuates HSV‐1 induced systemic inflammation [43]. The different and even controversial effects of Lachnospiraceae members on host well‐being might also be attributed to the differences of Lachnospiraceae members due to their diversities at species/strain levels and/or to their unique metabolisms.

As of the date of writing, the family Lachnospiraceae is comprised of validly published 80 genera and 176 species (https://lpsn.dsmz.de/family/lachnospiraceae) that originated from environments, humans, and animals. Still, many important Lachnospiraceae have neither been successfully cultivated nor described, and the uncultivated Lachnospiraceae members comprised almost 10% of the proposed prioritized 1468 gut microbial taxa [44]. Due to the limited resources of cultivated Lachnospiraceae strains from human guts, the metabolite pools of Lachnospiraceae are rarely explored, although several Lachnospiraceae species were characterized for productions of well‐known beneficial metabolites, such as SCFAs [45, 46, 47], vitamins [48], and pyrazine [49], or productions of harmful metabolites, such as cytotoxic and genotoxic *p*‐cresol [50].

Here, we report the cultivation and profiling of metabolites of Lachnospiraceae strains from healthy human adults. By modification of culturing methods, we newly cultured 114 Lachnospiraceae strains. Together with our previous Lachnospiraceae culture collections [51, 52], we collected 77 species representing 33 genera of the Lachnospiraceae family, and provided taxonomic descriptions of nine novel species and five genera (*h*uman‐originated *L*a*ch*nospiraceae *sp*ecies [hLchsp], https://hgmb.nmdc.cn/subject/lachnospiraceae). In total 242 metabolites comprised 17 major categories were detected for Lachnospiraceae strains (https://hgmb.nmdc.cn/subject/lachnospiraceae/metabolites). By evaluating the prevalence of the Lachnospiraceae metabolites in human fecal volatile‐metabolomic data sets, we found that 17 Lachnospiraceae metabolites were prevalent in human feces, and two of which were specifically enriched in nonalcoholic fatty liver disease (NAFLD) cohorts.

## RESULTS

### Cultivation and collection of Lachnospiraceae strains and the establishment of human‐derived Lachnospiraceae (hLchsp) biobank

**Figure 1:**
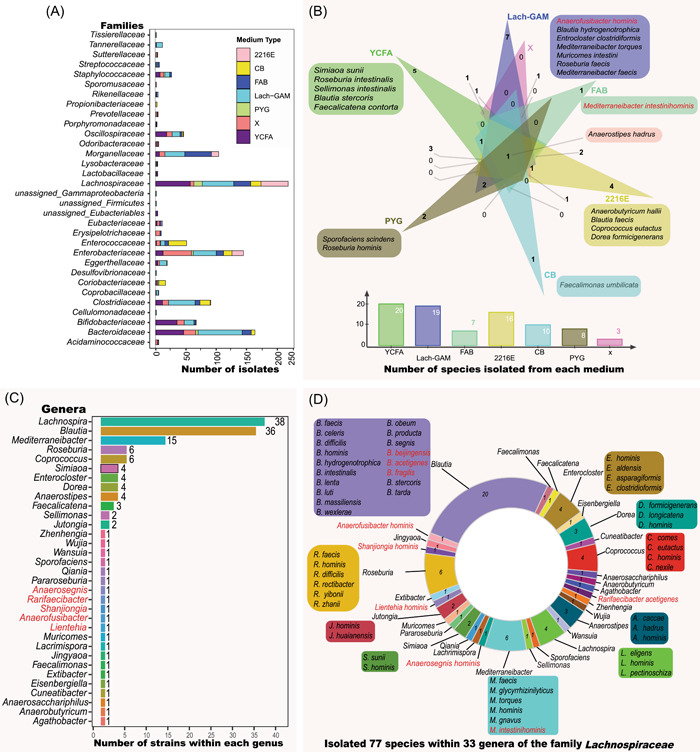
Cultivation and collection of Lachnospiraceae isolates for the hLchsp biobank. (A) Distribution at the family level of 1116 bacterial isolates. (B) Uniqueness at the species level of Lachnospiraceae growth on seven culture media. Panels (C) and (D) describe the features of the established hLchsp biobank. (C) Genus names and number of strains of each genus. (D) Species composition of hLchsp biobank. Numbers in the donut chart represent numbers of the species, and species names are provided outside the donut chart when a genus comprises more than one species. Red names represent novel taxa that are newly described in this study. hLchsp, *h*uman‐originated *L*a*ch*nospiraceae *sp*ecies; Lach‐GAM, Lachnospiraceae Gifu Anaerobic medium.

Matching culture medium components with bacterial physiology is critical to optimize bacterial cultivation. Thus, we explored the cultivation and physiological information of previously cultivated Lachnospiraceae strains. We first referred to the growth medium components of previously successfully cultivated 138 Lachnospiraceae species (Supporting Information Table S1A) that were listed in the Bacterial Diversity Metadatabase [53]. Analysis of the data sets revealed that 66 nonredundant media with different medium components were used to cultivate these 138 Lachnospiraceae strains. The most frequently applied components used as carbon and energy sources were cellobiose, maltose, starch, casitone, trypticase peptone, peptone, and glucose (Supporting Information Figure S1A). Second, we extracted the metabolic features of Lachnospiraceae strains from API 32A test results (Supporting Information Table S1C). Analysis revealed that most of the API 32A test results (*n* = 89 in total) were positive for *α*‐galactosidase (*n* = 58), *β*‐galactosidase (*n* = 65), *β*‐glucosidase (*n* = 45), and *α*‐arabinosidase (*n* = 42) (Supporting Information Figure S1B). Third, we investigated the carbon sources assimilation by 23 Lachnospiraceae species that were cultivated and characterized by BIOLOG test in our previous study [52], and found that the following substrates were frequently used by the Lachnospiraceae strains, that is, d‐galactose, *α*‐d‐glucose, l‐rhamnose, palatinose, l‐fucose, d‐fructose, d‐galacturonic acid, pyruvic acid, glyoxylic acid, 3‐methyl‐d‐glucose, d‐mannose, dextrin, d‐melibiose, glucose 6‐phosphate, and methyl pyruvate as the preferred carbon sources for the cellular growth (Supporting Information Figure S1C). Integrating the results above, we defined a new growth medium for the cultivation of Lachnospiraceae, namely, Lach‐GAM, by supplementing diet‐fiber‐derived carbohydrates into the Gifu anaerobic medium (GAM) [54]. The Lach‐GAM and additional six media‐ yeast casitone fatty aacids broth (YCFA), X media, Columbia blood agar (CB), fastidious anaerobe broth (FAB), peptone yeast glucose broth (PYG), and 2216E media Supporting Information Table S2) were applied for the cultivation of Lachnospiraceae from five fecal samples of healthy Chinese adults, following our previously established workflow [38]. Bacterial colonies were picked and purified by plate‐streaking, and further phylogenic associations were determined based on sequenced 16S RNA gene identities. In total, we obtained 1116 bacterial isolates (Supporting Information Table S3B) belonging to 32 families, and the top four families were Lachnospiraceae (219 isolates, 19.6%), Bacteriodaceae (164 isolates, 14.7%), Enterobacteriaceae (141 isolates, 13.0%), and Morganellaceae (104 isolates, 9.3%) (Figure 1A). Overall, we recovered diverse bacterial isolates, including over 30 Lachnospiraceae genera, which suggested the effectiveness of the current cultivation strategy regarding taxonomic diversity of Lachnospiraceae isolates. In the current study, by the long period of cultivation (>30 days), we recovered slow‐growing taxa, such as Coprococcus, Exibacter, and Eisenbergiella [55, 56, 57]. There were 57, 52, 46, 28, 6, 17, and 13 Lachnospiraceae isolates recovered from YCFA, Lach‐GAM, FAB, CB, PYG, 2216E, and X media, respectively (Figure 1B). There was overlapping of the species of cultivated Lachnospiraceae isolates from the seven media, and seven species (*Anaerofusibacter homins* gen. nov. sp. nov., *Blautia hydrogenotrophica*, *Entrocloster clostridiformis*, *Mediterraneibacter torques*, *Muricomes intestini*, *Roseburia faecis*, and *Mediterraneibacter faecis*) were recovered only from the newly defined Lach‐GAM medium. Five species (*Blautia stercoris*, *Faecalicatena contorta*, *Simiaoa sunii*, *R. intestinalis*, and *Sellimonas intestinalis*) are only from YCFA medium, and the four species (*A. hallii*, *Blautia faecis*, *Coprococcus eutactus*, and *Dorea formicigenerans*) only from 2216E medium, two species (*Sporofaciens scindens* and *R. homins*) only from PYG medium. *Mediterraneibacter intestinihomins* gen. nov. sp. nov. and *Faecalimonas umbilicata* were from FAB and CB media, respectively (Figure 1B). These results demonstrated that the Lach‐GAM medium was effective for growing Lachnospiraceae, and the application of multiple culture media facilitated the recovery of different taxa of Lachnospiraceae.

From the 219 Lachnospiraceae isolates, we successfully maintained and deposited 114 strains at China General Microbiological Culture Collection Centre (CGMCC). We also retrieved 34 strains from the Human Gut Microbiomes [52]. Integrating these Lachnospiraceae strains, we established the hLchsp biobank of 148 human‐derived Lachnospiraceae strains (https://hgmb.nmdc.cn/subject/lachnospiraceae, Supporting Information Table S3A, Figure 1C). The hLchsp biobank was composed of 77 species and 33 genera (including nine novel species and five novel genera that were firstly isolated, identified, and described in this study, see *Taxonomic descriptions of novel taxa*) within the family Lachnospiraceae (Figure 1D). The genera Lachnospira, Blautia, and Roseburia were prevalent inhabitants of human gastrointestinal (GI) with high abundances [1, 9], and they were well represented in the hLchsp biobank. There were 39 strains of *Lachnospira*, 36 strains of Blautia, and six strains of Roseburia (Figure 1C). Fifteen strains from genus Mediterraneibacter, including species of *M. faecis*, *Mediterraneibacter glycyrrhizinilyticus*, *M. torques*, *Mediterraneibacter hominis*, *M. gnavus*, *Mediterraneibacter intestinihominis*, were also covered. Additional members were from species of *Anaerosacchariphilus hominis*, *A. hallii*, *Anaerostipes caccae*, *Anaerostipes hadrus*, *Anaerostipes hominis*, *Coprococcus comes*, *C. eutactus*, *Coprococcus hominis*, *Coprococcus nexile*, *Cuneatibacter caecimuris*, *D. formicigenerans*, *Dorea longicatena*, *Dorea hominis*, *Eisenbergiella tayi*, *Enterocloster hominis*, *Enterocloster aldensis*, *Enterocloster asparagiformis*, *Enterocloster clostridioformis*, *Extibacter muris*, *F. contorta*, *F. umbilicata*, *Lacrimispora celerecrescens*, *M. intestini*, *S. intestinalis*, and *S. scindens* (Figure 1D and Supporting Information Table S3A). So far as we know, this is the first targeted collection of Lachnospiraceae strains.

### Lachnospiraceae strains produce diverse metabolites

**Figure 2:**
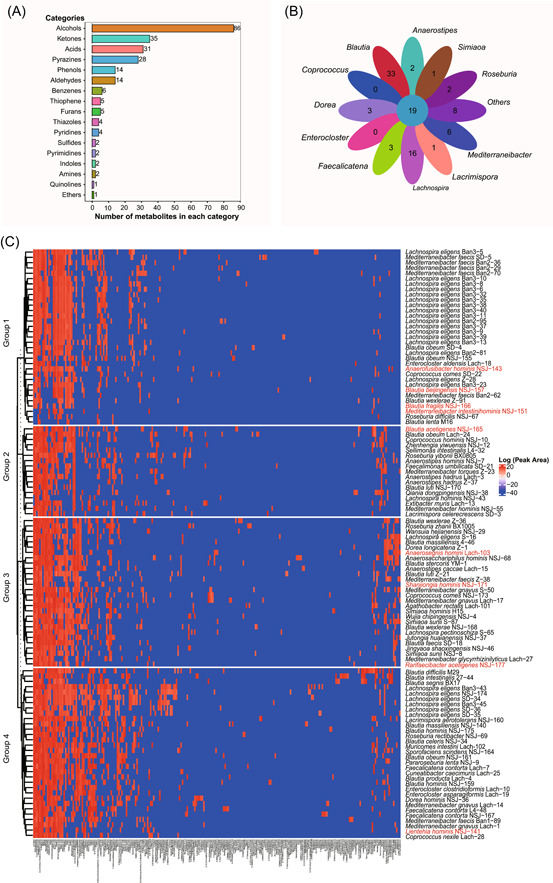
Profiling of metabolites from 110 strains of Lachnospiraceae. (A). Catalogories of 242 metabolites. (B) Shared and unique metabolites among genera of Lachnospiraceae. The inner circle denotes the shared 19 metabolites, and each leaf represents a genus group. The numbers shown on leaves represent genus‐specific metabolites. (C) Heatmap of metabolites from 110 Lachnospiraceae strains. Color describes the relative amounts of metabolites represented with the Log values of peak area; blue to red indicates relative amounts from low to high, strain names in red denoting novel taxa.

We cultivated all 148 hLchsp Lachnospiraceae strains and found that 110 strains successfully grew in the Lach‐GAM broth. The grown cultures were extracted and proceeded for metabolite profiling, while the metabolites in the sterile Lach‐GAM medium measured together with inoculated bacterial cultures were included as a blank control, and any metabolite detected in the control was removed from the metabolite profiles of bacterial cultures. Totally 242 nonredundant metabolites were identified (for detailed metabolites of each strain, refer to Supporting Information Table S4A) and they were classified into 17 categories according to chemical natures. The top categories were alcohols (89), ketones (35), pyrazines (29), acids (31), phenols (14), and aldehydes (14) (Figure 2A).

We identified metabolites shared or uniquely featured to Lachnospiraceae members at the genus level (Figure 2B). Results showed that there are 19 shared metabolites at the genus level. They were acetic acid, butyric acid, hexadecanoic acid, octadecanoic acid, ethanol, *n*‐dodecanol, *n*‐hexanol, *n*‐tetradecanol, pentanol, skatole, 2,5‐dimethyl‐4‐hydroxy‐3(2H)‐furanone, *p*‐cresol, 2,5‐dimethylpyrazine, 2,5‐methylethylpyrazine, 2,6‐dimethylpyrazine, 5‐isopentyl‐2,3‐dimethylpyrazine, methylpyrazine, pyrazine, and trimethylpyrazine. Noticeably, 2,5‐dimethyl‐4‐hydroxy‐3(2H)‐furanone, a general plant‐originated antimicrobial agent, was reported to be produced by *Zygosaccharomyces rouxii* [58, 59], and there has been no report about the production by prokaryotes so far. We would contain the interpretation of genus‐shared metabolites within those Lachnospiraceae strains, as many of the genera were represented by only a couple of strains. Exceptions were the genera Blautia and Lachnospira that were well represented (36 and 38 strains, respectively). We found that the genera Blautia and Lachnospira produced more unique metabolites: The genus Blautia produced 33 (alcohols and acids), and the genus Lachnospira produced 16 (alcohols, ketones, and pyrazines) unique metabolites (Figure 2B).

We tried to correlate the metabolite profiles with bacterial phylogenies. As shown in Figure 2C, Spearman clustering [60] of metabolites yielded four groups. The members of group 1 were mainly Lachnospira strains and a few Mediterraneibacter and Blautia strains, and they mainly produced SCFAs, furanones, and alcohols, and they all produce propionic and butyric acids. The members of group 2 were composed of strains from Blautia, Mediterraneibacter, Agathobacter, and unclassified Lachnospiraceae strains. Bacterial strains of this group 2 mainly produced butyric and hexanoic acids, and the amounts of production were relatively high. Group 3, mainly composed of Blautia and several members of Mediterraneibacter, Anaerostipes, Coprococcus, and Roseburia, was the largest cluster that was metabolically highly active and their metabolites were diverse, including alcohols, acids, ketones, and aldehydes. Group 4 was composed of strains of Enterocloster, Dorea, Faecalicatena, Muricomes, Sporofaciens, and several members of Blautia, and members within this group were metabolically less active.

### Lachnospiraceae species are generally productive for SCFAs but are significantly different at the strain level

**Figure 3:**
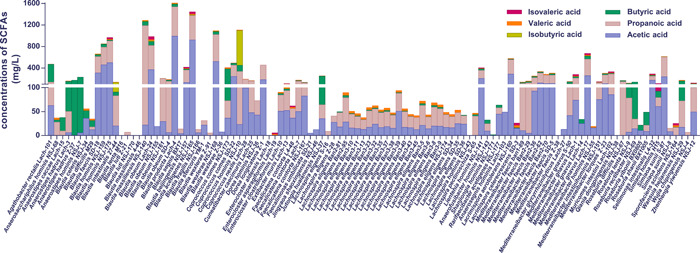
Production of SCFAs by Lachnospiraceae strains. Bars in violet, ochre, green, light green, orange, and red represent acetic, propionic, butyric, isobutyric, valeric, and isovaleric acids, respectively; in this scattered bar chart, the height of each bar in different color represents the amount (mg/L) of SCFAs production. SCFAs, short‐chain fatty acids.

SCFAs exert important probiotic functions on host health: Butyrate serves as the primary energy source for intestinal epithelial cells [61], and suppresses pathogen colonization [62]. Acetate can mediate fat accumulation [63] and can be converted into butyrate [64, 65]. Propionate was reported to lower the serum cholesterol levels of the host [66]. We examined the capabilities of 110 Lachnospiraceae strains for SCFAs production, and quantified their SCFAs production (Figure 3). Results showed that there were 91, 88, 78, and 73 Lachnospiraceae strains producing acetic, propionic, butyric, and valeric acids, respectively. Twenty‐four and 19 Lachnospiraceae strains produced also isobutyric and isovaleric acids, respectively. These results demonstrated that most of the Lachnospiraceae species particularly Blautia species were able to produce SCFAs. The productions of SCFAs with Lachnospiraceae species and strains are shown in Figure 3. Among the acetic acid producers, the top five strains were *B. producta* Lac‐4, *B. acetigenes* NSJ‐165, *B. wexlerae* NSJ‐168, *B. homins* NSJ‐175, and *B. homins* NSJ‐159, and their productions were 991.3, 923.4, 522.5, 498.8, and 459.4 mg/L after 3 days cultivation at 37°C in Lach‐GAM liquid medium, respectively. The top five Lachnospiraceae strains for propionic acid production were *B. massiliensis* NSJ‐140, *B. producta* Lach‐4, *B. wexlerae* NSJ‐168, *B. acetigenes* NSJ‐165, and *B. obeum* Lach‐24, and they produced 1165.5 and 547.1, 499.5, 451.0, and 48.9 mg/L, respectively. The top five Lachnospiraceae strains for butyric acid production were *A. rectalis* Lach‐101, *C. comes* NSJ‐173, *A. homins* NSJ‐7, *J. huaianensis* NSJ‐37, and *A. hadrus* Z‐37, and they produced 331.5, 310.9, 224.8, 186.9, and171.9 mg/L, respectively. Other strains that yield over than 50 mg/L butyric acids were *C. hominis* strain NSJ‐10, *R. rectibacter* strain NSJ‐69, *A. hadrus* strain Lach‐3, *W. hejianensis* strain NSJ‐29, *B. massiliensis* strain NSJ‐140, *B. obeum* strain Lach‐24, *B. hominis* strain NSJ‐159, *M. gnavus* strain Lach‐17, *B. wexlerae* strain NSJ‐168, and *B. producta* strain Lach‐4, indicating several few reported taxa also could be potential probiotics. The top five Lachnospiraceae strains for valeric acid production were *B. producta* Lach‐4, *C. comes* NSJ‐173, *B. acetigenes* NSJ‐165, *B. producta* Lach‐4, and *B. massiliensis* NSJ‐140, and they produced 21.1, 20.1, 14.9, 13.9, and 12.1 mg/L, respectively. The top five Lachnospiraceae strains for isobutyric acid production were *C. homins* NSJ‐10, *B. intestinalis* 27‐44, *B. obeum* Lach‐24, *B. massiliensis* NSJ‐140, and *B. acetigenes* NSJ‐165, and they produced 647.7, 42.1, 16.3, 14.4, and 11.3 mg/L, respectively. The top five Lachnospiraceae strains for isovaleric acid production were *B. obeum* Lach‐24, *B. acetigenes* NSJ‐165, *M. gnavus* Lach‐17, *B. homins* NSJ‐175, and *M. gnavus* Lach‐1, and they produced 56.9, 41.7, 33.2, 31.2, and 28.6 mg/L, respectively (Figure 3).

Although Lachnospiraceae strains were generally productive for SCFAs, significant differences occurred at species and strain levels in productivities and composition. Thus, we evaluated further the productions for SCFAs of Blautia (36 strains) and Lachnospira (38 strains). As shown in Figure 3, Blautia generally showed higher SCFAs production than Lachnospira at species and strain levels. Yet, very different productions of SCFAs among strains of Blautia or Lachnospira were observed (Figure 3). For example, *B. wexlerae* strain NSJ‐168 produced high amounts of acetic and propionic acids, but *B. wexlerae* strain Z‐36 did not produce significant amounts of SCFAs. *L. homins* NSJ‐43 did not produce SCFAs. The difference in SCFAs productions was also observed for members of other genera in the hLchsp biobank. The recently discovered *S. sunii* strain NSJ‐8 produced trace amounts, but *S. sunii* strain S‐87 produced high amounts, of acetic and propionic acids (Figure 3).

### Productions of alcohols (including farnesol derivatives), aldehydes, and ketones by Lachnospiraceae strains

**Figure 4:**
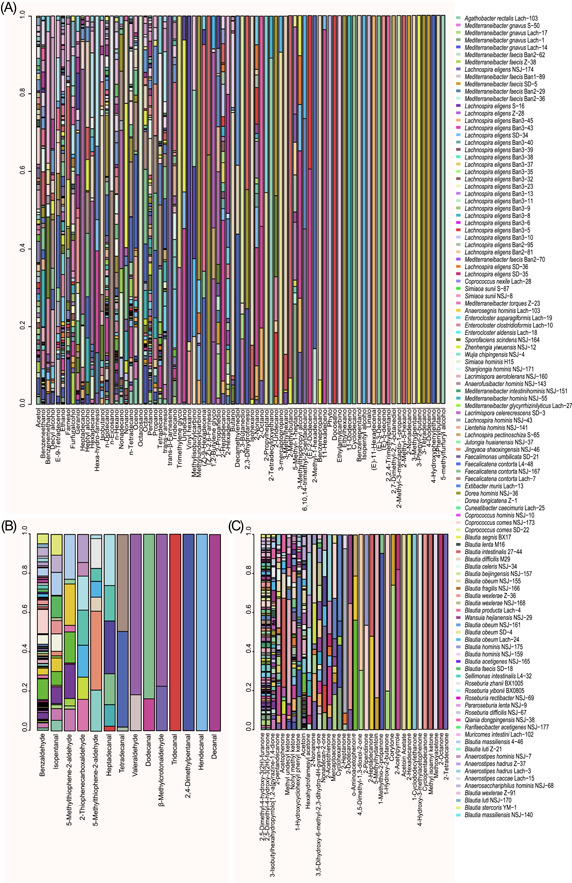
Productions of alcohols, aldehydes, and ketones by 110 Lachnospiraceae strains. (A) Scattered bar chart demonstrating the relative amounts of alcohols (*n* = 86) produced by 110 strains. (B) Scattered bar chart demonstrating the relative amounts of aldehydes (*n* = 14) produced by 110 strains. (C) Scattered bar chart demonstrating the relative amounts of ketones (*n* = 35) produced by 110 strains. And for panels (A)–(C), the relative amounts of each metabolite were represented by the relative percentage of metabolite GC‐MS peak area. GC‐MS, Gas Chromatography–Mass Spectrometry.

The productions of alcohols (C2–C19) were detected for the 110 Lachnospiraceae strains, and the results showed that strains of Blautia, Roseburia, Lachnospira, Muricomes, Faecalicatena, Mediterraneibacter, Enterocloster, Dorea, and Enterocloster were the major producers (Figure 4A). We observed that more than half of the 110 strains produced ethanol (*n* = 88), pentanol (*n* = 86), *n*‐dodecanol (*n* = 85), acetol (*n* = 73), *n*‐tetradecanol (*n* = 64), phenylethyl alcohol (*n* = 63), and *n*‐hexanol (*n* = 62). Importantly, certain alcohols were detected with known physiological functions to hosts but were not well investigated for gut microbial production. For example, we detected that 33, 36, and 3 *Lachnopiraceae* strains produced farnesol, trans‐farnesol, and 2,3‐dihydrofarnesol, respectively. Farnesol and its isomers or derivatives have been reported to be able to regulate host metabolisms and had anti‐inflammatory functions [67, 68]. Antimicrobial and antifungal alcohols were also produced by many of *Lachnopiraceae* strains, such as geraniol (*n* = 26) [69], nonyl alcohol (*n* = 17) [70], 2‐undecanol (*n* = 6) [71], decyl alcohol (*n* = 23), and 1‐octanol (*n* = 3) (Supporting Information Table S4B).

**Figure 5:**
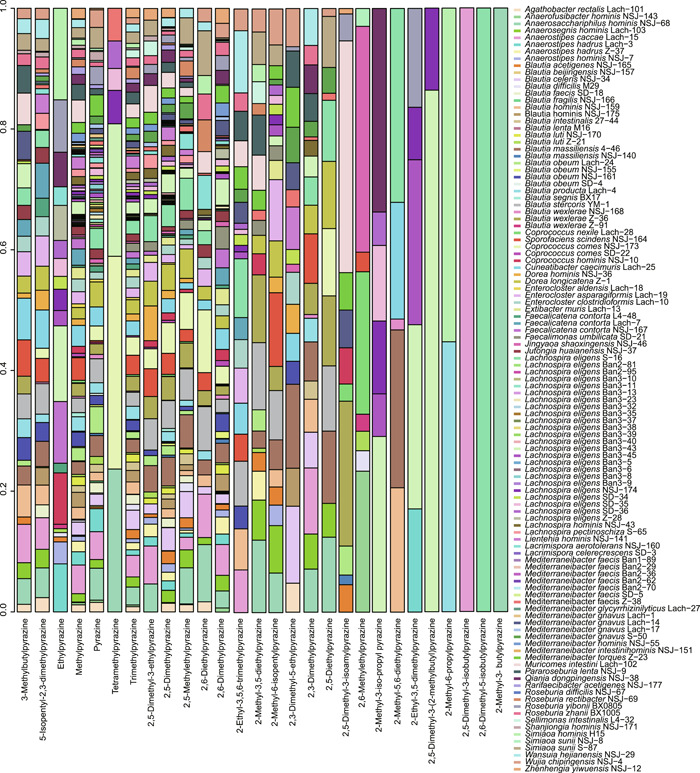
Productions of pyrazine and its derivatives by the 110 Lachnospiraceae strains. The scattered bar chart demonstrates the relative amounts of pyrazines produced by 110 strains (represented by the relative percentage of metabolite GC‐MS peak area). GC‐MS, Gas Chromatography–Mass Spectrometry.

Compared with the beneficial effects of alcohols, aldehydes are generally considered to trigger oxidizing stresses and are harmful to hosts [72]. Our results (Figure 4B) showed that Lachnospiraceae strains produced a range of aldehydes, for example, benzaldehyde (*n* = 91), isopentanal (*n* = 18), 5‐methyl‐2‐thiophenecarboxaldehyde (*n* = 10), 3‐methyl‐2‐thiophenecarboxaldehyde (*n* = 8), *cis*‐9‐hexadecenal (*n* = 6), heptadecanal (*n* = 6), 2,4‐dimethylbenzaldehyde (*n* = 7), pentanal (*n* = 3), dodecanal (*n* = 2), 2,4‐dimethylpentanal (*n* = 1), (*e*)‐11‐hexadecenal (*n* = 1), decanal (*n* = 1), tridecanal (*n* = 1), and undecanal (*n* = 1). The 14 aldehydes produced by Lachnospiraceae strains are presented in Figure 5, and we found that some of the robust alcohol producers were also active in aldehyde production, such as strains of the genera Blautia, Lacrimispora, Roseburia, Anaerostipes, Mediterraneibacter, Sellimonas, Anaerosacchariphilus, Lachnospira, Dorea, and Coprococcus.

Surprisingly, ketones were also widely produced by Lachnospiraceae strains (Figure 4C). The frequently produced ketones were 2,5‐dimethyl‐4‐hydroxy‐3(2H)‐furanone (*n* = 98), acetoin (*n* = 60), and 3‐isobutylhexahydropyrrolo[1,2‐*a*]pyrazine‐1,4‐dione (*n* = 45) that was reported to be an antifungal active agent against dermatophytes and filamentous fungi [73, 74, 75, 76]. The following ketones were also detected, they were nonyl methyl ketone (*n* = 37), 1‐hydroxycyclohexyl phenyl ketone (*n* = 23), 3,5‐dihydroxy‐6‐methyl‐2,3‐dihydro‐4h‐pyran‐4‐one (*n* = 16), methyl undecyl ketone (*n* = 14), acetophenone (*n* = 12), hexahydrofarnesyl acetone (*n* = 9), mercaptoacetone (*n* = 9), 2‐acetothienone (*n* = 8), 2‐nonanone (*n* = 8), nonadecan‐2‐one (*n* = 7), cyclohexanone (*n* = 5), 2‐heptanone (*n* = 4), 2‐dodecanone (*n* = 3), *o*‐aminoacetophenone (*n* = 3), 1‐hydroxy‐2‐butanone (*n* = 2), 1‐methylthio‐2‐propanone (*n* = 2), 2‐acetylpyrrole (*n* = 2), 2‐heptadecanone (*n* = 2), 2‐piperidinone (*n* = 2), 4,5‐dimethyl‐1,3‐dioxol‐2‐one (*n* = 2), 5‐methylhydantoin (*n* = 2), and corylone (*n* = 2). The top producers of ketones were strains of Roseburia, Lachnospira, Faecalimonas, Zhenhengia, Enterocloster, Lacrimispora, Mediterraneibacter, Wansuia, Qiania, Blautia, Coprococcus, and Extibacter (Figure 4C).

### Production of pyrazine and derivatives by Lachnospiraceae strains

Pyrazines and their derivatives are of great pharmaceutical importance and have been developed as antimicrobial and antifungal drugs [77, 78]. To our surprise, the production of pyrazines and their derivatives were frequently observed in the tested 110 Lachnospiraceae strains (Figure 5). For example, 106 strains produced 2,5‐dimethylpyrazine and methylpyrazine, 90 strains produced trimethylpyrazine, and 83 strains produced pyrazine. Interestingly, a previously reported effective metabolite with nervous activity 2,3‐dimethylpyrazine (also named 3DP) [79] was detected in 21 Lachnospiraceae strains. Tetramethylpyrazine, a documented agent with antioxidation and anti‐inflammatory functions and a metabolite that ameliorated hepatic fibrosis [80] and protects mice retinas against oxidative injury, and it was reported to be produced by *Bacillus coagulans* [81], and we found that it was also produced by seven Lachnospiraceae strains, namely, *M. faecis* strain Z‐38 and *L. eligens* strain NSJ‐174, *L. eligens* strain Ban3‐43, *L. eligens* strain SD‐36, *L. eligens* strain SD‐35, *C. comes* strain NSJ‐173 and *A. homins* strain NSJ‐68. There were Lachnospiraceae strains (*n* = 23) produced 2‐ethyl‐3,5‐dimethyl pyrazine that was produced also by *E. coli* strains [82]. There were 23 Lachnospiraceae strains that produced the flavoring additive 2‐ethyl‐3,5, 6‐trimethyl pyrazine (*n* = 23), which was reported to be produced by *Bacillus* [83]. Lachnospiraceae strains *L. eligens* strain SD‐35 and *M. torques* strain Z‐23 produced trimethylpyrazine that showed antibacterial activities against the pathogenic *Staphylococcus aureus* [84].

As shown in Figure 5, many Lachnospiraceae strains produced more than one pyrazine and derivatives. The major productive strains of Lachnospiraceae were from the following genera, Enterocloster, Dorea, Faecalicatena, Blautia, Simiaoa, Lachnospira, Cuneatibacter, Anaerosacchariphilus, Coprococcus, Muricomes, Sporofaciens, and Anaerostipes.

### Major metabolites produced by Blautia and Lachnospira strains

**Figure 6:**
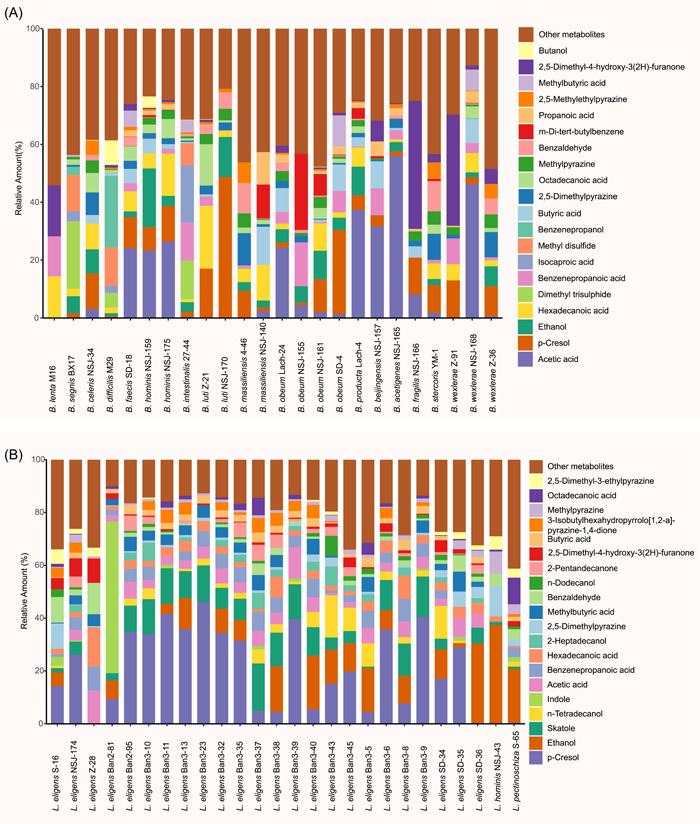
Major metabolites produced by Blautia (A) and Lachnospira (B) strains. The heights of bar segments represent relative amounts of metabolites (represented by GC‐MS peak area), and only the top 20 metabolites were shown. GC‐MS, Gas Chromatography–Mass Spectrometry.

Being highly abundant and prevalent bacteria in the gut, Blautia and Lachnospira attracted more attention for their probiotic or harmful roles in host health [46, 85]. We further explored their metabolite productivities. Figure 6A,B shows the top 20 metabolites from Blautia and Lachnospira strains. The major and common metabolites produced by Blautia were butanol, 2,5‐dimethyl‐4‐hydroxy‐3‐2h‐furanone, methylbutyric acid, 2,5‐methylethylpyrazine, propanoic acid, *m*‐di‐*tert*‐butylbenzene, benzaldehyde, methylpyrazine, octadecanoic acid, 2,5‐dimethylpyrazine, butyric acid, benzenepropanol, methyl disulfide, isocaproic acid, benzenepropanoic acid, dimethyl trisulphide, hexadecanoic acid, ethanol, *p*‐cresol, and acetic acid (Figure 6A).

Unlike Blautia, the strains of Lachnospira showed similar features in producing metabolites, but the amounts of metabolites were relatively low, and we found that 2,5‐dimethyl‐3‐ethylpyrazine, octadecanoic acid, methylpyrazine, 3‐isobutylhexahydropyrrolo‐12‐*a*‐pyrazine‐14‐dione, 2‐pentandecanonene, dodecanol, 2,5‐dimethyl‐4‐hydroxy‐3(2H)‐furanone, benzaldehyde, methylbutyric acid, 2‐heptadecanol, hexadecanoic acid, benzenepropanoic acid, acetic acid, indole, *n*‐tetradecanol, skatole, ethanol, and *p*‐cresol were produced by most of the strains within genus Lachnospira (Figure 6B).

### Distribution and prevalence of Lachnospiraceae‐derived metabolites in human cohorts

**Figure 7:**
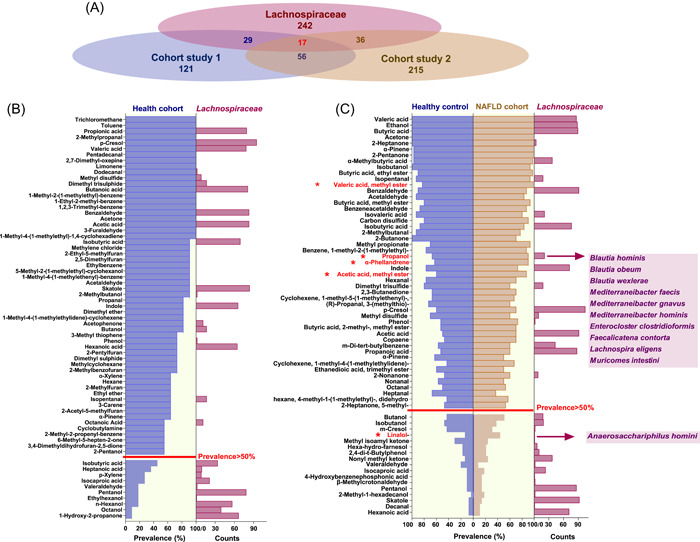
Distribution and prevalence of Lachnospiraceae‐derived metabolites in human fecal samples of different cohorts. (A) The Venn diagram demonstrating the coverage of volatile metabolites in different cohort studies by Lachnospiraceae metabolites from this study. Cohort study 1 comprising fecal samples from healthy humans (*n* = 11). Cohort study 2 comprising fecal samples from nonalcoholic fatty liver disease (NAFLD) cohort (*n* = 30) and its healthy counterparts (*n* = 30). (B) Bar charts displaying the prevalent volatile metabolites in fecal samples of healthy humans from Cohort study 1 (violet bars) and the numbers of Lachnospiraceae producers in this study (wine red bars). (C) Bar charts displaying the prevalent volatile metabolites in fecal samples of Cohort study 2 (healthy control *n* = 30, violet bars; and NAFLD patients *n* = 30, ochre bars) and the numbers of Lachnospiraceae producers in this study (wine red bars). The red asterisk marked five metabolites that were significantly enriched in NAFLD cohort, and two of which were identified in this study (the Lachnospiraceae producers are shown in the panel).

To determine the distribution and prevalence of volatile metabolites produced in vitro by Lachnospiraceae strains in real‐world human GI environments, we extracted and reanalyzed the volatile metabolome data sets of two cohort‐based studies (Cohort study 1 [86] and Cohort study 2 [87]). As shown in Figure 7A, 121 metabolites were identified from 11 fecal samples of the healthy cohort (Cohort study 1), 29 of which were recovered from the Lachnospiraceae‐produced metabolites. For Cohort study 2, 215 volatile metabolites were characterized from fecal samples of 30 NAFLD patients and 30 healthy controls, and 36 of the detected fecal metabolites were recovered from the Lachnospiraceae‐produced metabolites. All recovered metabolites as well as the numbers of Lachnospiraceae producers are displayed in Figure 7B,C. Notably, there were only 56 volatile metabolites shared by both studies, while 17 were covered by the Lachnospiraceae‐produced metabolites, accounting for 30% of the in‐common fecal metabolites (Figure 7A). We then investigated the prevalence of Lachnospiraceae‐produced metabolites in human cohorts. If we define a metabolite with a prevalence >50% among fecal samples in each study as “prevalent,” 58 and 49 metabolites were identified as prevalent fecal metabolites for Cohort studies 1 and 2, respectively. As shown in Figure 7B,C, 33% and 39% of the volatile metabolites prevailing in human feces were produced by Lachnospiraceae strains from this study. Noteworthily, as previous studies reported that Lachnospiraceae were specifically enriched in the gut microbiota of NAFLD patients [87], we further evaluated if the Lachnospiraceae‐produced metabolites were enriched in the NAFLD cohort. There were five metabolites specifically enriched in NAFLD cohort, compared with the healthy control groups, and two of them (1‐propanol and 1,6‐octadien‐3‐ol,3,7‐dimethyl) were produced by Lachnospiraceae species as shown in Figure 7C.

## DISCUSSION AND CONCLUSIONS

In this study, we established a human Lachnospiraceae (hLchsp) biobank and profiled Lachnospiraceae metabolite. The establishment of the hLchsp biobank benefitted from the improvement of oriented cultivation of Lachnospiraceae species. Previous studies have demonstrated that GAM medium can be used to cultivate many of the prevalent and abundant obligate gut anaerobes, including members of Oscillospiraceae, Clostridiaceae, Bacteroidaceae, and Lachnospiraceae [50, 54], and that YCFA and FAB media are used to cultivate aerointolerant gut bacteria [56, 88, 89, 90]. By integrating current knowledge of cultivation and the physiology of Lachnospiraceae, we developed a new medium, namely, Lach‐GAM, from the GAM medium. By using Lach‐GAM and other six culture media, including YCFA and FAB media, a range of Lachnospiraceae species and strains were cultivated, including four newly nominated genera and nine novel species (refer to the description of novel taxa in the Material and methods section). Considering that there are many slow‐growing microorganisms, we extend the culturing period to 30 days by using a variety of culture media, and as a result, we recovered certain slow‐growing taxa such as Coprococcus, Exibacter, and Eisenbergiella that were rarely isolated during the previous Lachnospiraceae targeted cultivation‐based study [57]. Our efforts on oriented cultivation significantly increased the previous collections of 27 Lachnospiraceae species [57], and the hLchsp biobank has 148 strains of 77 species, covering 33 genera within the family Lachnospiraceae. Our results indicated that the modified culture methods and various culture media can effectively recover diverse intestinal microbiota, which is also consistent with the previous reports [89, 91, 92]. In addition, some of the Lachnospiraceae species covered in this study were reported to affect host health. For example, *B. producta* showed the ability to inhibit lipid accumulation and effectively ameliorated hyperlipidemia [93]; a strain of *R. hominis* increases intestinal melatonin level [19]. *A. rectalis* suppresses lymphomagenesis [42] and attenuates HSV‐1 induced systemic inflammation [43]; and a strain of Anaerostipes was reported to have beneficial roles in renal function [94]. These reports indicate that the Lachnospiraceae biobank we established will provide a broad range of research materials for related studies, and will facilitate any follow‐up researches regarding functions and mechanisms of these species or strains.

With Gas Chromatography–Mass Spectrometry (GC‐MS) and solid‐phase microextraction (SPME)/GC‐MS methods, we determined 242 metabolites from 110 Lachnospiraceae strains, and we quantified the productions of the SCFAs (C2–C5). Our results showed that besides the members of previously well‐acknowledged probiotic genera Roseburia [95, 96, 97], strains of genera Coprococcus, Blautia, Anaerostipes, Agathobacter, and Jutongia also produce considerably high amounts of butyric acid, a metabolite that could improve host immunity and regulate tissue inflammation [98], and thus these strains could be considered novel and potential probiotics for further exploration. Blautia was reported to exert beneficial and harmful impacts on host health by different studies [46, 99, 100]. We searched for the previous studies about SCFAs production of Lachnospiraceae, and found that *C. nexile* KCTC 5578, *M. torques* ATCC 27756, *Faecalicatena fissicatena* KCTC, and some Blautia and *F. umbilicata* members produce acetate [101, 102, 103, 104], while *C. comes* ATCC 27758 and some Roseburia and Enterocloster members produce butyrate [105, 106, 107]. In this study, we found that different Blautia strains produced diverse metabolites of unknown bioactivities, and exerted different abilities in the production of butyric acids. We noticed that butyrate production by the Roseburia also strain‐specific. Our study would provide bacterial resources and metabolites that support future investigations of host–microbiome interactions at either bacterial species or strain levels.

In addition, it is noteworthy that even though there are numerous reports about the beneficial effects of SCFAs mentioned above, the potentially adverse or contradictory effects of SCFAs on host health were also reported [108, 109]. High concentrations of SCFAs inhibited the growth of pathogens, such as Salmonella and *S. aureus* [110, 111, 112], but low levels of propionate as a carbon source facilitated the growth of Salmonella [113]. Therefore, the role of SCFAs should be evaluated carefully, as the conclusions reached in different studies may be SCFAs type and concentration dependent.

Besides SCFAs, we also detected many other metabolites from Lachnospiraceae strains, which might play important roles in the human intestines. Hexadecanoic and octadecanoic acids, both bactericidal active compounds [114], were produced by 106 Lachnospiraceae strains. Farnesol and its isomers or derivatives have been reported to have antipathogenic, anti‐inflammatory, and antifungal functions, which are critical to host health [115, 116, 117, 118]. Our results showed that the Mediterraneibacter and Lachnospira strains produced farnesol of different amounts, and these strains could be prioritized in further studies concerning host–microbiome and microbe–microbe interactions. Geraniol has anti‐*Candida* activity via disruption of cell membrane integrity and function [119, 120]. We found that geraniol was produced by the abundant gut inhabitants including Blautia, Lachnospira and Mediterraneibacter strains in this study, which clued their potential roles in the modulation of pathogenic fungi.

Correlating to the reported harmful effects of Lachnospiraceae on host health, we also detected Lachnospiraceae metabolites that are toxic or trigger host dysbiosis. *p*‐Cresol, a reported toxin with cytotoxicity and genotoxicity and reduced endothelial barrier function [121, 122, 123], was frequently detected in this study (*n* = 109). Phenol was another frequently detected metabolite in this study, and it was a reported tumor‐promoting agent [124]. Skatole, being a gut microbial catabolite of tryptophan and able to elicit AhR‐mediated death of intestinal epithelial cells [125, 126], was also frequently detected in this study. There were many functionally unknown metabolites from the 110 Lachnospiraceae strains. Benzaldehyde (*n* = 91), trimethylpyrazine (*n* = 90), pentanol (*n* = 86), and *n*‐dodecanol (*n* = 85) were the frequently detected ones. These metabolites are apparently harmful at higher concentrations to host health, but their involvement in interactions of host–microbiome and microbe–microbes would be worthy of further investigations.

However, our present study also has some limitations. We determined SCFAs both by referring to the NIST11 library and standards, while other substances were determined by referencing the NIST11 library only. We have detected that the Lachnospiraceae produced diverse metabolites, including pyrazines, ketones, and phenols in vitro, which were seldom reported to be produced by gut microbes in previous studies [127, 128]. For further interests concerning these metabolites, further standards‐based validations are necessary. Especially for those metabolites that were rarely identified as microbial products, such as pyrazine derivatives, including 2,3‐dimethylpyrazine and 2,5‐dimethyl‐4‐hydroxy‐3(2H)‐furanone, both of which were reported to be generated by the Maillard reaction of plant‐based substances [79, 129], further GC‐MS analysis with standards as well as in silico analysis of the potential genes or pathways involved in their production at genome level would enable a better understanding of the metabolism and functional potentials of Lachnospiraceae in gut microbiota.

## MATERIALS AND METHODS

### Human feces sample collection and pretreatment

The whole project was approved by the Research Ethics Committee of the Institute of Microbiology, Chinese Academy of Science (ethical approval No. APIMCAS2017049). All the donors of fecal samples were enquired about their health conditions, history of clinical visits for the last half‐year, and history of antibiotic treatments for the last 2 months in person before a consent form was signed for the donation of feces. Five adults (ages ranging from 24 to 33) from Beijing, China without any clearly diagnosed chronic and malignant disease were considered healthy donors, and their feces samples were collected using sterile tubes and placed onto ice packs, transferred into the Electrotek Anaerobic Workstation (AW 400SG) filled with CO_2_/H_2_/N_2_ (5%/10%/85%) gas mix for further use.

### Culture media

The growth factors, medium components, and carbon source utilization of the previously cultured Lachnospiraceae strains were collected from publications and public data sources (Supporting Information Table S1A). We obtained the growth medium component data of nonrepeated 66 media used for culturing 138 strains from 66 species of 18 genera within the family Lachnospiraceae, and 32A enzyme data sets of 103 bacterial species. The media used in this study and their components are listed in Supporting Information Table S1C. The transfer and distribution of broth and agar media were conducted at anaerobic conditions under 100% nitrogen flow and media were autoclaved at 115°C for 25 min.

### Bacterial isolation, cultivation, and storage

The bacterial isolation, cultivation, and storage were performed as described in our previous studies [52]. Briefly, the fecal samples were washed, pelleted, and suspended three times with 0.01 M of phosphate buffer solution (PBS, pH 7.4) (Cat No. P1022, Solarbio Com. Ltd.) before filtration using 40 μm cell sieves (FALCON) for removal of insoluble particles. The filtration was serially diluted (10^−1^−10^−7^) with anoxic PBS supplemented with peptone (0.2% w/v) and l‐cysteine, and the appropriate dilutions (10^−4^–10^−7^) were spread on the agar plates of different culture media for incubation at 37°Cfor 2–30 days, anaerobically. The reason for incubation for such a long period is to recover the slow‐growing and less‐abundant, and any yet‐to‐be cultured bacteria taxa [55, 56]. Single colonies that appeared on the plates were picked, and 16S rRNA genes were sequenced by Tianyi Huiyuan Co. Ltd., and the targeted pure cultures were transferred into the liquid broth for further experimentation and onto agar slopes in Hungate tubes for long‐term storage. Colonies on agar slope were washed with 15% (v/v) glycerite for cryopreservation at −80°C. All operations were conducted in the anaerobic workstation unless otherwise indicated.

### Bacterial identification and characterization of novel taxa

The cultured bacterial strains were sequenced by Tianyi Huiyuan Co. Ltd. for 16S rRNA gene using the universal primers 27f and 1492r [19], and searched for close relatives using EzBioCloud [20]. For all Lachnospiraceae strains, nearly full‐length 16S rRNA gene sequences were generated and are listed in Supporting Information Table S3A. The delineations of novel taxa were based on the analysis of each type of strain in terms of phylogenetic, genomic, physiological, and morphological characteristics as described in our previous works [62, 130], and the criteria used for the proposal of novel species/genus/family described in our previous publication [52]. In brief, thresholds of 98.7% and 94.5% 16S rRNA gene sequence identities were considered as indications for novel species and genera, respectively [39]. The digital DNA: DNA hybridization (dDDH) values <70% and average nucleotide identity (ANI) values <95% were considered as an indication for separate species [64, 69]. The percentage of conserved proteins (POCPs) values <50% was considered as an indication for separate genera [66]. The dDDH values were calculated with Genome‐to‐Genome Distance Calculator 2.0 at http://ggdc.dsmz.de [64]. The ANI values were calculated with OrthoANI [65]. The POCPs were determined using BLASTP (thresholds for delineation of aligned sequences: *E*‐value <1e^−5^, identity >40%, and query coverage of >50%) [66]. The 16S rRNA gene‐based phylogenetic trees and genome‐based phylogenomic trees of newly isolated strains and their related type species were created using UBCG [67], and presented in Supporting Information Figures S2A–8A and Figures S2B–8B. The bacterial cell morphology was observed using a transmission electron microscope JEM‐1400 (JOEL) (Supporting Information Figures S2C–S8C). The nomenclature of each characterized novel taxa was proposed according to the rules of the International Code of Nomenclature of Prokaryotes [131] The descriptions of novel taxa were presented below and in Supporting Information Data 1.

### Descriptions of novel taxa

Anaerofusibacter gen. nov. (An.ae.ro.fu.si.bac'ter. Gr. pref. *an*‐, not; Gr. masc. n. *aer* (gen. *aeros*), air; n. *fusus*, a spindle; N.L. masc. n. *bacter*, rod; N.L. masc, anaerobic spindle‐shaped rod bacteria). The genus *Anaerofusibacter* is a member of the Lachnospiraceae family. The type species is *A. homins*.

*A. homins* sp. nov. (ho'mi.nis. L. gen. masc. n. *homins*, of human origin, refers to the type strain isolated from human fecal samples). The type strain NSJ‐143 (=CGMCC 1.17904) was isolated from the feces of a healthy adult. The genome size of the type strain is 2.67 Mbp, and the G + C content is 41.51 mol%. Cells are spindle‐shaped (0.6–1.2 µm wide by 1.2–2.6 µm long), Gram‐positive, non‐spore‐forming, and nonmotile. Cells grow under strictly anaerobic conditions and appear singly or in pairs. After 72 h of anaerobic incubation at 37°C, tiny, smooth white colonies appeared on the Lach‐GAM medium. Cells utilize d‐cellobiose, d‐fructose, l‐fucose, d‐galactose, d‐galacturonic acid, gentiobiose, d‐glucosamine, *α*‐d‐glucose, glucose‐6‐phosphate, d‐mannose, d‐melibiose, 3‐methyl‐d‐glucose, palatinose, l‐rhamnose, turanose, uridine, lactulose, and maltose for growth. The major cellular fatty acids are C_16:0_ and C_18:0_. The major polar lipids are unknown phospholipids, lipids, and glycolipids.

Anaerosegnis gen. nov. (A.nae.ro.sge.nis. Gr. pref. *an*‐, not; Gr. masc. n. *aer (gen. aeros)*, air; L. masc./fem. adj. *segnis*, slow. a slow [growing] anaerobic organism). The genus *Anaerosegnis* is a member of the Lachnospiraceae family. The type species is *Anaerosegnis homins*.

*A. homins* sp. nov. (ho'mi.nis. L. gen. masc. n. *homins*, of a man, the host from which the species was first isolated). The type strain Lach‐103 (=CGMCC 1.46159) was isolated from the feces of a healthy Chinese adult. The genome size of the type strain is 3.03 Mbp and the G + C content is 42.49 mol%. Cells are rod‐shaped, Gram‐negative, straight rods, nonmotile, and non‐spore‐forming. Cells grow under strictly anaerobic conditions and appear singly. Growth occurs at the pH range of 6.5–7.5 (optimum 7.0), a temperature range of 35–40°C (optimum 37°C). Tiny, slightly raised, transparent colonies appear on Lach‐GAM agar plates after 6 days of incubation at 37°C. Cells utilize d‐cellobiose, d‐fructose, l‐fucose, d‐galactose, d‐galacturonic acid, gentiobiose, d‐glucosaminic acid, *α*‐d‐glucose, glucose‐6‐phosphate, maltose, d‐mannose, d‐melibiose, 3‐methyl‐d‐glucose, palatinose, l‐rhamnose, turanose, uridine, *α*‐d‐lactose, lactulose, and propionic acid for growth.

*B. acetigenes* sp. nov. (a.ce.ti'ge.nes.L. neut. n. *acetum*, acetic acid; Gr. suff.‐*genes*, forming; from L. v. *gigno*, to form; N.L. part. adj. *acetigenes*, acetogenic, refers to the bacterium produces acetic acid). The type strain NSJ‐165 (CGMCC 1.17923) was isolated from the feces of a healthy adult. The genome size of the type strain is 6.46 Mb, and the G + C content is 46.00 mol%. Cell size is 0.4–0.8 µm × 0.6–1.5 µm. Cells grow under strictly anaerobic conditions and are neither spore‐forming nor motile. The optimal growth pH is 6.5–7.5, and the optimal growth temperature is 30–37°C. After 2 days at 37°C, round (0.5–1.1 mm in diameter), dry, flat, yellow to white, rough‐edged colonies appear on Lach‐GAM agar plates. Cells utilize d‐cellobiose, d‐fructose, l‐fucose, d‐galactose, d‐galacturonic acid, gentiobiose, d‐glucosamine, *α*‐d‐glucose, glucose‐6‐phosphoric acid, maltose, d‐mannose, d‐melibiose, 3‐methyl‐d‐glucose, palatinose, l‐rhamnose, turanose, and uridine, *α*‐d‐lactose, lactose, fructose, and glyoxylic acid for growth. The major cellular fatty acid components are C_16:0_, C_14:0_, and C_17:0 2OH_.

*Blautia beijingensis* sp. nov. (bei.jing.en'sis. N.L. fem. adj. *beijingensis*, of Beijing, in Beijing, specifically refers to the strain isolated in Beijing). Type strain NSJ‐157 (=CGMCC 1.17918) was isolated from the feces of a healthy adult. The genome size of the type strain is 3.61 Mb, and the G + C content of genomic DNA is 44.08 mol%. Cells are oval to short rod‐shaped (0.2–0.6 µm × 0.5–1.2 µm), non‐spore‐forming and nonmotile. Cells grow under strictly anaerobic conditions and mostly occur in pairs or single. The colony is round, raised, and moist on Lach‐GAM agar plates after 2 days of incubation at 37°C. Growth occurs at a pH of 6.5–7.5 (optimum 7.0), and a temperature of 30–45°C (optimum 37°C). Cells utilize d‐arabitol, d‐cellobiose, erythritol, d‐fructose, l‐fucose, d‐galactose, d‐galacturonic acid, gentiobiose, d‐gluconic acid, d‐glucosamine, *α*‐d‐glucose, 6‐phosphoglucose, inositol, lactulose, d‐mannose, d‐melibiose, 3‐methyl‐d‐glucose, palatinose, l‐rhamnose, turanose, glyoxylic acid, pyruvate and methyl pyruvate, dextrin, and succinic acid for growth.

*Blautia fragilis* sp. nov. (fra'gi.lis. L. fem. adj. *fragilis*, fragile, Cells are fragile and hard to culture). The type strain NSJ‐166 (CGMCC 1.46116) was isolated from the feces of a healthy adult. The genome size of the type strain is 3.61 Mb and genomic DNA G + C content is 44.03 mol%. Cells are oval to short rod‐shaped (0.7–1.1 µm × 1.4–2.1 µm), non‐spore‐forming and nonmotile. Cells grow anaerobically and appear singly or in pairs. The growth occurs at pH of 6.5–7.5 (optimum 6.5), and a temperature of 30–37°C (optimum 7.0). After 4 days of anaerobic incubation at 37°C, tiny, slightly raised clear colonies (0.8–1.2 mm in diameter) appeared on Lach‐GAM agar plates. Cells utilize d‐fructose, l‐fucose, d‐galactose, d‐galacturonic acid, gentiobiose, d‐glucosamine, *α*‐d‐glucose, Lactulose, d‐mannose, 3‐methyl‐d‐glucose, palatinose, l‐rhamnose, turanose, glyoxylic acid, pyruvate, glucose‐6‐phosphate, *m*‐inositol, *α*‐ketobutyric acid, *α*‐ketovaleric acid, and methyl pyruvate for growth.

Lientehia gen. nov. (Lien.teh'i.a. N.L. fem. n. *Lientehia*, named in honor of Wu Lienteh, a famous Chinese microbiologist and medical scientist who has made great contributions to the fight against the plague). Genus *Lientehia* is a member of the family Lachnospiraceae. The type species is *Lientehia homins*.

*L. homins* sp. nov. (ho'mi.nis. L. gen. masc. n. *homins*, of a human being, referring to the human gut habita). The type strain NSJ‐141 (=CGMCC 1.17902=KCTC 25345) was isolated from the feces of a healthy Chinese adult. The genome size of the type strain is 3.05 Mbp and the G + C content is 48.7 mol%. Cells are fusiform (0.5–0.8 µm wide and 0.9–1.2 µm long), Gram‐negative, nonmobile and non‐spore‐forming. Cells grow anaerobically and appear in dividing pairs or in dividing chains. Growth occurs at the temperature of 30–45°C (optimum 37°C), pH of 6–7.5 (optimum pH, 6.5), and NaCl pressure of 0%–3.0% (w/v). After incubation at 37°C for 72 h, yellow, smooth, circular, or irregular, flat colonies with semitransparent extended margins in a diameter of 0.9–1.5 mm appear on modified gifu anaerobic medium agar medium. Cells metabolize adonitol, d‐cellobiose, dextrin, d‐fructose, l‐fucose, d‐galactose, d‐galacturonic acid, *α*‐d‐glucose, glucose‐6‐phosphate, lactulose, d‐mannose, d‐melibiose, 3‐methyl‐d‐glucose, palatinose, l‐rhamnose, turanose, glyoxylic acid, pyruvic acid and pyruvic acid methyl ester, d‐arabitol, d‐gluconic acid, d‐glucosaminic acid, *m*‐inositol, and maltose for growth. The predominant cellular fatty acids are C_16:0_ and C_18:0*ω*7c_. The major polar lipids are diphosphatidylglycerol, phosphatidylglycerol, phosphatidylglycolipids, unidentified phospholipids, and glycolipids.

*M. intestinihomins* sp. nov. (in.tes.ti.ni.ho'mi.nis. L. gen. neut. n. *intestini*, intestinal; L. gen. masc. n. *homins*, of human, N.L. gen. masc. n. *intestinihomins*, human intestinal, refers to type strains isolated from human intestinal). The type strain NSJ‐151 (=CGMCC 1.17913) was isolated from the feces of an adult. The genome size is 3.41 Mbp, and the G + C content of the genomic DNA is 39.0 mol%. Cells are fusobacterium (0.6–1.0 µm × 1.2–2.3 µm), Gram‐positive, non‐spore‐forming, and nonmotile. Cells utilize d‐fructose, l‐fucose, d‐galactose, d‐galacturonic acid, gentiobiose, *α*‐d‐glucose, 6‐phosphoglucose, d‐mannose, d‐melibiose, 3‐methyl‐d‐glucose, palatinose, l‐rhamnose, glyoxylic acid, pyruvate, d‐cellobiose, d‐glucosamine, lactulose, turanose, and l‐asparagine for growth. The major cellular fatty acids are C_16:0_, C_17:0 2OH_, and C_14:0_. Glucose metabolism produces acetate, propionate, isobutyrate, and butyrate.

Rarifaecibacter gen. nov. (Ra.ri.fae.ci.bac.ter L. masc. adj. *rarus*, rare; fae.ci. L. masc. n. *faex* (gen. *faecis*), pertaining to feces; N.L. masc. n. *bacter*, a rod, a rarely studied bacterium isolated from stool, named because this species is rarely isolated). The genus Rarifaecibacter is a member of the Lachnospiraceae family. The type species is *Rarifaecibacter acetigenes*.

*R. acetigenes* sp. nov. (a.ce.ti'ge.nes. L. neut. n. *acetum*, vinegar; Gr. suff.‐genes, *producing*; from Gr. ind. v. *gennaô*, to produce; N.L. part. adj. acetigenes, vinegar‐ or acetic acid‐producing). Type strain NSJ‐177 (=CGMCC 1.17905) was isolated from the feces of a healthy adult. The genome size of the type strain is 4.28 Mbp and the G + C content is 52.08 mol%. Cells are rod‐shaped (0.6–0.9 µm × 1.8–2.4 µm), Gram‐positive, non‐spore‐forming, and nonmobile. After 48 h of anaerobically incubation at 37°C, tiny, raised gray colonies were formed on the Lach‐GAM medium. Cells utilize ribitol, d‐cellobiose, d‐fructose, l‐fucose, d‐galactose, d‐galacturonic acid, gentiobiose, *α*‐d‐glucose, 6‐phosphate glucose, *α*‐d‐lactose, lactulose, d‐mannose, d‐melibiose, 3‐methyl‐d‐glucose, palatinose, l‐rhamnose, turanose, glyoxylic acid, pyruvate, methyl pyruvate, d‐arabitol, and dextrin for growth. The major cellular fatty acids are C_16:0_ and C_14:0_. The major polar lipids of cells are diphosphatidylglycerol, phosphatidylglycerol, and phosphatidylethanolamine as well as unknown lipids.

Shanjiongia gen. nov. (Shan.jiong.i'a. N.L. fem. n. *Shanjiongia*, named in honor of the famous microbiologist Shen Shanjiong). The genus *Shanjiongia* is a member of the Lachnospiraceae family. The type species is *Shanjiongia homins*.

*S. homins* sp. nov. (ho'mi.nis. L. gen. masc. n. *homins*, human, referring to the type strain was isolated from human fecal samples). Type strain NSJ‐171 (=CGMCC 1.17927) was isolated from the feces of a healthy adult. The genome size is about 2.35 Mbp, and the G + C content is about 33.0 mol%. Cells are oval‐shaped, Gram‐negative, non‐spore‐forming, and nonmotile. Cells grow under strictly anaerobic conditions and appear singly or in pairs. Cells utilize erythritol, d‐fructose, l‐fucose, d‐galactose, d‐galacturonic acid, gentiobiose, d‐glucosamine, *α*‐d‐glucose, lactulose, maltose trisaccharide, d‐mannose, d‐melibiose, 3‐methyl‐d‐glucose, palatinose, l‐rhamnose, turanose, pyruvate, glucose‐6‐phosphate, glyoxylic acid, *α*‐ketobutyric acid, and methyl pyruvate for growth. The major cellular fatty acids are C_14:0_ and C_16:1_.

### Determination of SCFAs

The concentrations of SCFAs (including acetate, propionate, butyrate, valerate, isobutyrate, and isovalerate) were determined using GC‐MS. Bacterial cells were incubated at 37°C anaerobically in Lach‐GAM broth for 72–168 h until OD_600 nm_ reached 1.0–1.2, then the cells were collected. For in vitro detection of SCFAs production, no SCFA was added in the liquid medium. The cell cultures were measured for each strain, and the sterile liquid medium was used as a blank control in which no SCFA peak was detected by GC‐MS analysis. According to Sumner et al., the SCFAs identified in this study belonged to level 1‐identified compounds, which referenced both standards and the NIST library [132]. For each sample, 1 ml cell culture was extracted with 1 ml ethyl acetate, and the supernatant was prepared for GC‐MS analysis performed on a GCMS‐QP2010 Ultra with an autosampler (SHIMADZU) and the DB‐wax capillary column (30 m, 0.25 mm i.d., 0.25 μm film thickness, SHIMADZU). Standard curves of SCFAs were achieved by pure chemical agents of corresponding chemicals, purchased from Aladdin, diluted in ethyl acetate of chromatographic purity, dilution rate, and corresponding peak area data were detailed in Supporting Information Table S4A. The temperature of the oven was programmed from 35°C to 130°C at 5°C/min gradients, to 230°C at 30°C/min gradients, with 16 min hold. Injection of 2 μl samples was performed at 230°C. The carrier gas, helium, flowed at 1.0 ml/min. Ion source and interface temperature were both set at 230°C. The electronic impact was recorded at 70 eV.

### Profiling of metabolites with SPME and GC‐MS

All 110 Lachnospiraceae strains were profiled for metabolites with GC‐MS after SPME. Sterile, noninoculated Lach‐GAM medium was used as control. The SPME fiber, 50/30 μm DVB/CAR/PDMS, Stableflex (Supelco), was inserted through the septum of cell‐culture tubes and exposed in the headspace of the vial for 60 min, to allow complete absorption of the volatile compounds onto the SPME fiber. The SPME fiber was then introduced into the injector port of the gas chromatograph for 1 min in splitless mode, injection temperature was set at 240°C, to desorb the volatile compounds. Helium was used as a carrier gas with a flow of 1.0 ml/min and the oven temperature was programmed as follows: 40°C for 3 min, then ramped at 5°C/min to 240°C, held for 15 min. Ion source and interface temperature were both set at 240°C. The metabolites were identified by searching the obtained mass spectrum in the National Institute for Technology Standards (NIST11; www.nist.gov) mass spectral library with a threshold match score >85 rather than comparison with standards, and according to Sumner et al., the metabolites identified in this study belonged to level 2‐identified compounds, which referenced the NIST library only [132]. Data were reported as the peak area for each compound detected. Spectrum search encompasses baseline subtraction and averaging over a peak. Similar to the determination of SCFAs, the sterile Lach‐GAM liquid medium was taken as blank control, and peaks detected in the corresponding blanks were eliminated from the metabolite profiles of bacterial cultures, for the obtainment of signals attributed solely to bacterial metabolic activity. Relative amounts of metabolites were presented by the relative percentages of peak areas of metabolites produced by each strain. Average peak intensities were mean‐centered and unit‐scaled. All the processes of data analysis and visualization were conducted by using the ggplot2 package [23], RColorBrewer [68], and complex‐heatmap package in R [24].

## AUTHOR CONTRIBUTIONS

Shuang‐Jiang Liu and Chang Liu designed and supervised the entire study. Rashidin Abdugheni, Wen‐Zhao Wang, Yu‐Jing Wang, Meng‐Xuan Du, Feng‐Lan Liu, Cheng‐Ying Jiang, Nan Zhou, and Chang‐Yu Wang conducted the experiments. Linhuan Wu and Juncai Ma constructed the websites for hLchsp. Rashidin Abdugheni drafted the manuscript. Shuang‐Jiang Liu and Chang Liu revised, edited, and finalized the manuscript. All the authors confirmed the final version.

## CONFLICT OF INTEREST

The authors declare no conflict of interest.

## ETHICS STATEMENT

The ethics application (APIMCAS2017049) was approved by the Research Ethics Committee of the Institute of Microbiology, Chinese Academy of Sciences.

## Supporting information

Supporting information.

Supporting information.

## Data Availability

The data were available at the National Microbiology Data Center (NMDC, https://nmdc.cn/) under accessions NMDC10018179. Moreover, the information of all 148 strains and their 16S rRNA gene sequences were accessible via the hLchsp website (https://hgmb.nmdc.cn/subject/lachnospiraceae), and the in vitro metabolite profiles of 110 Lachnospiraceae strains were available at https://hgmb.nmdc.cn/subject/lachnospiraceae/metabolites. Supporting Information materials (figures, tables, scripts, graphical abstract, slides, videos, Chinese translated version, and update materials) may be found in the online DOI or iMeta Science http://www.imeta.science/.
