# Supplementary material for: Metabolite profiling of human‐originated Lachnospiraceae at the strain level
Source: Imeta. 2022 Oct 13;1(4):e58. doi: 10.1002/imt2.58 (PMC10989990; doi:10.1002/imt2.58)
Supplement: Supplementary file 1 — Supporting information. [file IMT2-1-e58-s002.docx]

Supplementary materials

**Metabolite profiling of human-originated *Lachnospiraceae* at strain levels**

Rashidin Abdugheni^1,4#^, Wen-Zhao Wang^3#^, Yu-Jing Wang^1,5^, Meng-Xuan Du^2^, Feng-Lan Liu^1,6^, Nan Zhou^1^, Cheng-Ying Jiang^1,5^, Chang-Yu Wang^7^, Linhuan Wu^1^, Juncai Ma^1^, Chang Liu^2*^, Shuang-Jiang Liu^1,2,5*^

^1^State Key Laboratory of Microbial Resources, and Environmental Microbiology Research Center (EMRC), Institute of Microbiology, Chinese Academy of Sciences, Beijing, 100101, P. R. China;

^2^State Key Laboratory of Microbial Technology, Shandong University, Qingdao 266000, P. R. China;

^3^State Key Laboratory of Mycology, Institute of Microbiology, Chinese Academy of Sciences, Beijing, 100101, P. R. China;

^4^State Key Laboratory of Desert and Oasis Ecology, Xinjiang Institute of Ecology and Geography, Chinese Academy of Sciences, Urumqi, 830011, P.R. China;

^5^University of Chinese Academy of Sciences, Beijing 100049, P. R. China;

^6^College of Life Sciences, Hebei University, Baoding, 071000, P. R. China;

^7^University of Science and Technology of China, Hefei, 230026, P.R. China;

*Authors for correspondence:

Shuang-Jiang Liu ([liusj@im.ac.cn](mailto:liusj@im.ac.cn)); Chang Liu, [liu.c@sdu.edu.cn](mailto:liu.c@sdu.edu.cn)

Institute of Microbiology, Chinese Academy of Sciences

NO.1 Beichen West Road, Chaoyang District, Beijing 100101, China

Tel: +86-10-64807423; Fax: +86-10-64807421

# These authors equally contributed to this work.

**Abbreviations:** SCFAs: short-chain fatty acids; GC-MS: gas chromatography mass spectrometry; SPME, Solid phase microextraction; Microbiome metabolomics.

**Keywords: *Lachnospiraceae*; Cultivation; *Blautia*; *Lachnospira*; Metabolite; Short Chain Fatty Acids (SCFAs);**


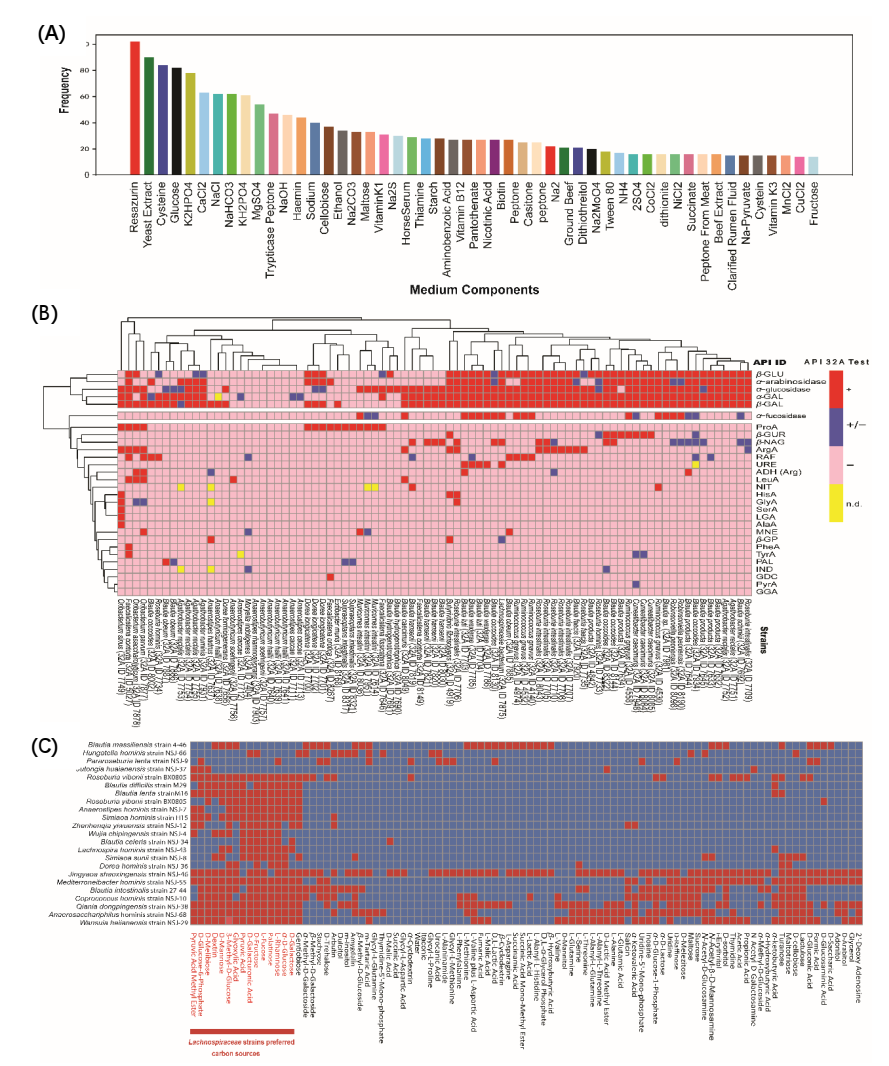


**Figure S1.** Visualization of meta-analysis results for optimization of *Lachnospiraceae* culture medium. **(A)** culture medium components used for cultivation of 138 *Lachnospiraceae* strains. (B) metabolic enzyme features of 89 *Lachnospiraceae* strains (represented by API 32A test results). The data were extracted from the Bacterial Diversity Metadatabase (<https://bacdive.dsmz.de/>) and are provided as supplementary datasets. **(C)** the carbon sources assimilation by the 23 *Lachnospiraceae* strains previously cultivated and reported [45];


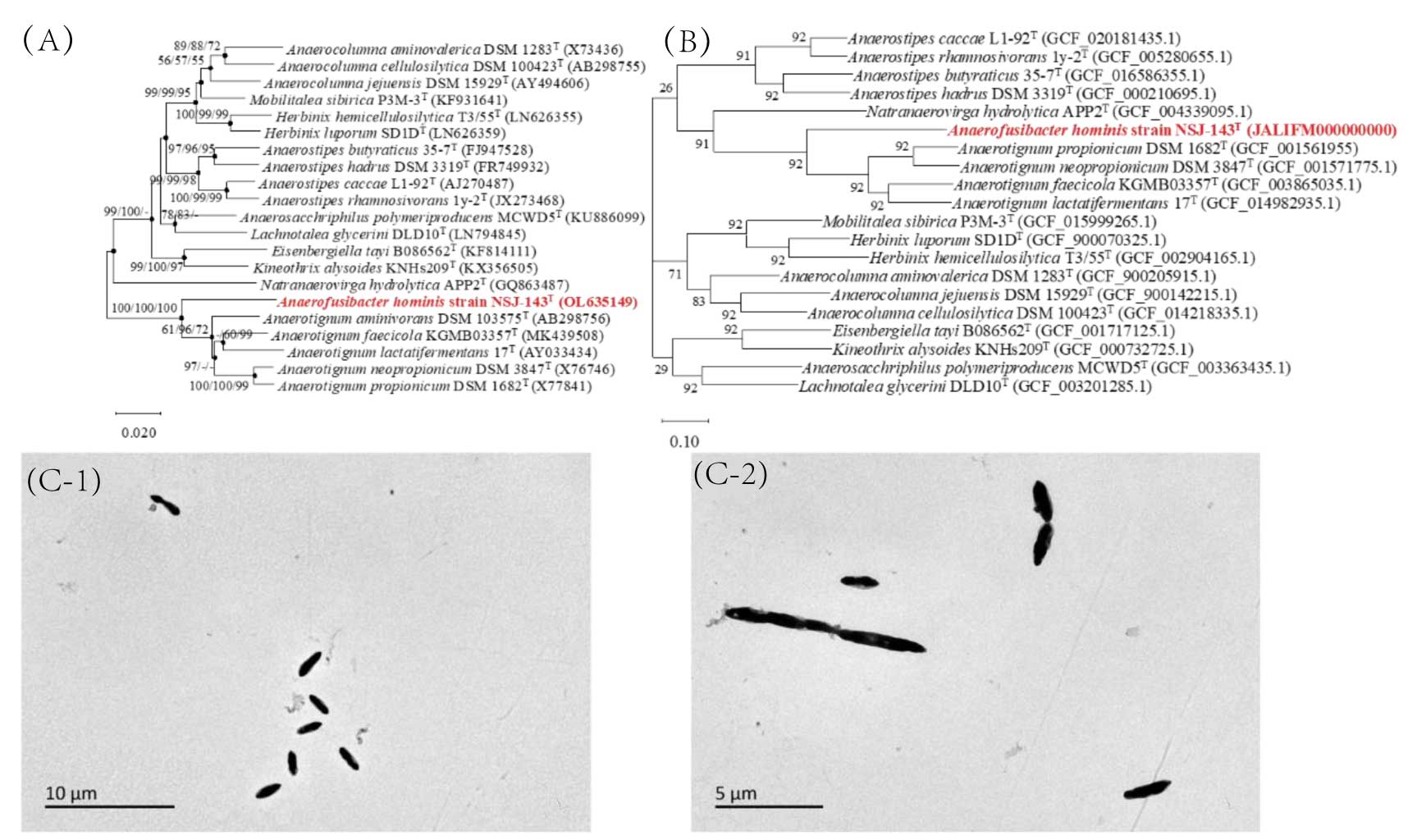


**Figure S2.** (A) Phylogenetic tree based on nearly complete 16S rRNA gene sequences showing the relationship between strain NSJ-143^T^ and its phylogenetic neighbors. This tree was constructed by using maximum-likelihood method with K2+G+I type with 1,000 bootstraps, phylogenetic trees based on the maximum-parsimony and the neighbour-joining methods with 1,000 bootstraps were also cunstructed, and bootstrap values (percentage of replicates) above the threshold of ≥50 % are shown for those nodes supported in at least one of the three methods; these bootstrap values are depicted in the order NJ/ML/MP. Filled circles indicate nodes reconstructed by all three methods. GenBank accession numbers are given in parentheses. Bar, 0.02 substitutions per nucleotide position; (B) Phylogenomic tree of strain NSJ-143^T^ and closely related strains based on 92 bacterial core gene sequences constructed using Up-to-date bacterial core genes (UBCG) of the genomes of related strains available on NCBI GenBank. GenBank accession numbers are shown in parentheses. Gene Support Index (GSI) values of 92 UBCGs are given at branching points; (C-1, C-2) Transmission electron micrograph of NSJ-143^T^ cells grown on Lach-GAM agar.

^
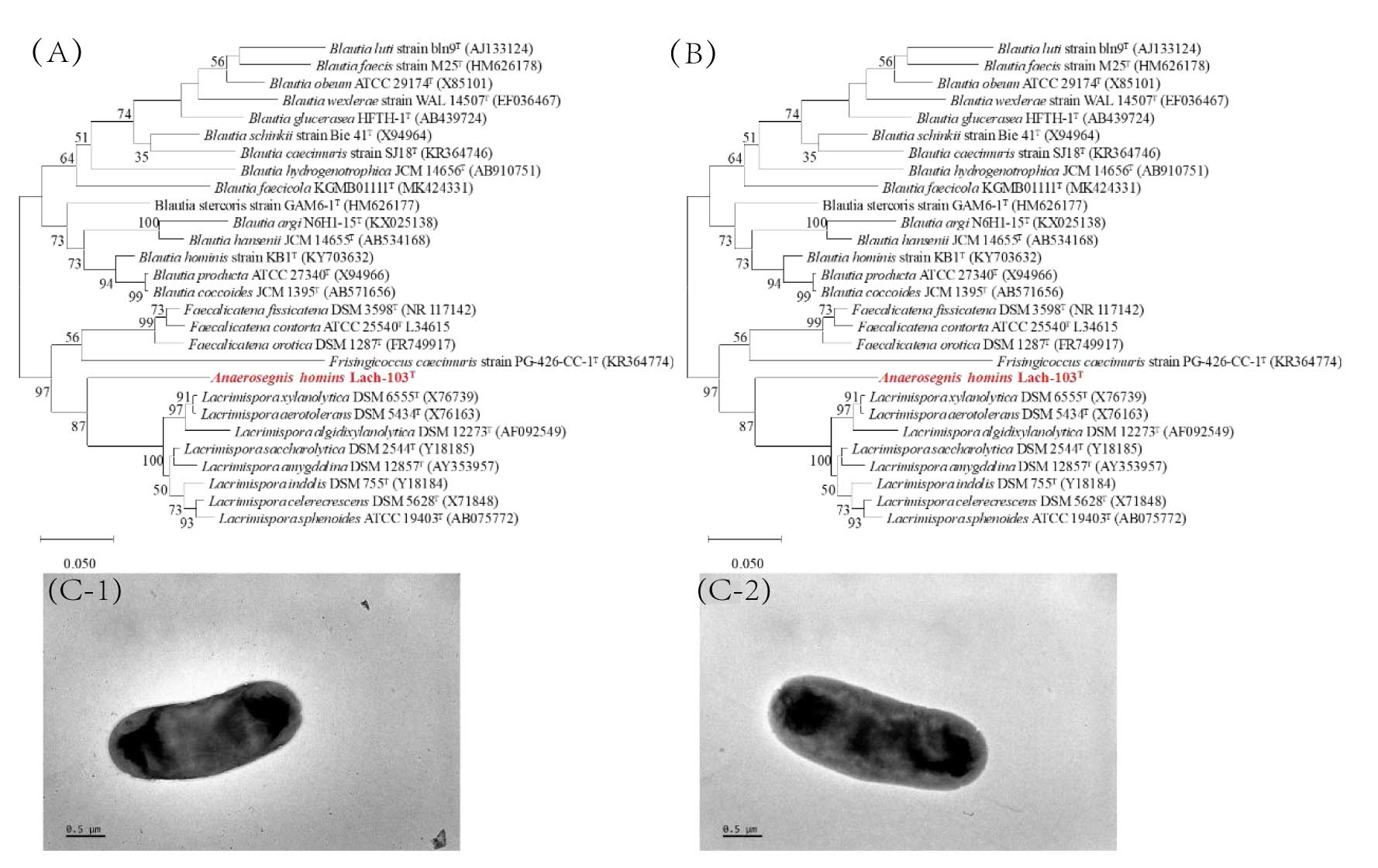
^

**Figure S3.** (A) Phylogenetic tree based on nearly complete 16S rRNA gene sequences showing the relationship between strain Lach-103^T^ and its phylogenetic neighbors. This tree was constructed by using maximum-likelihood method with K2+G+I type with 1,000 bootstraps, and bootstrap values above the threshold of ≥50 % are shown. GenBank accession numbers are given in parentheses. Bar, 0.05 substitutions per nucleotide position; (B) Phylogenomic tree of strain Lach-103^T^ and closely related strains based on 92 bacterial core gene sequences constructed using Up-to-date bacterial core genes (UBCG) of the genomes of related strains available on NCBI GenBank. GenBank accession numbers are shown in parentheses. Gene Support Index (GSI) values of 92 UBCGs are given at branching points; (C-1, C-2) Transmission electron micrograph of Lach-103^T^ cells grown on Lach-GAM agar.


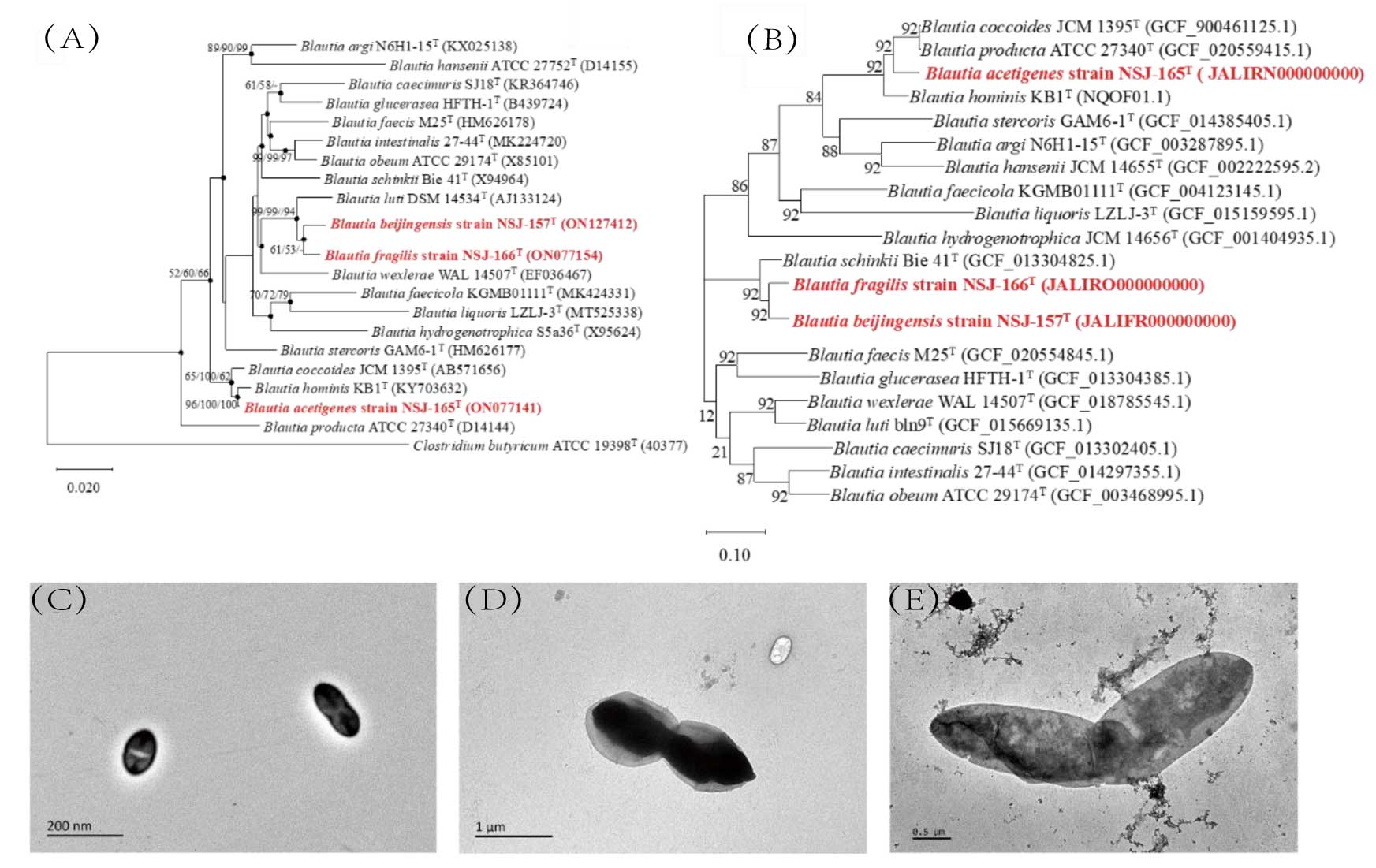


**Figure S4.** (A) Phylogenetic tree based on nearly complete 16S rRNA gene sequences showing the relationship between strain NSJ-157^T^, NSJ-165^T^ and NSJ-166^T^ and its phylogenetic neighbours. This tree was constructed by using maximum-likelihood method with K2+G+I type with 1,000 bootstraps, phylogenetic trees based on the maximum-parsimony and the neighbour-joining methods with 1,000 bootstraps were also cunstructed, and bootstrap values (percentage of replicates) above the threshold of ≥50 % are shown for those nodes supported in at least one of the three methods; these bootstrap values are depicted in the order NJ/ML/MP. Filled circles indicate nodes reconstructed by all three methods. GenBank accession numbers are given in parentheses. Bar, 0.02 substitutions per nucleotide position; (B) Phylogenomic tree of strain NSJ-157^T^, NSJ-165^T^ and NSJ-166^T^ and closely related strains based on 92 bacterial core gene sequences constructed using Up-to-date bacterial core genes (UBCG) of the genomes of related strains available on NCBI GenBank. GenBank accession numbers are shown in parentheses. Gene Support Index (GSI) values of 92 UBCGs are given at branching points;**(C, D, E)** Transmission electron micrograph of NSJ-157^T^, NSJ-165^T^ and NSJ-166^T^ cells grown on Lach-GAM agar, respectively.


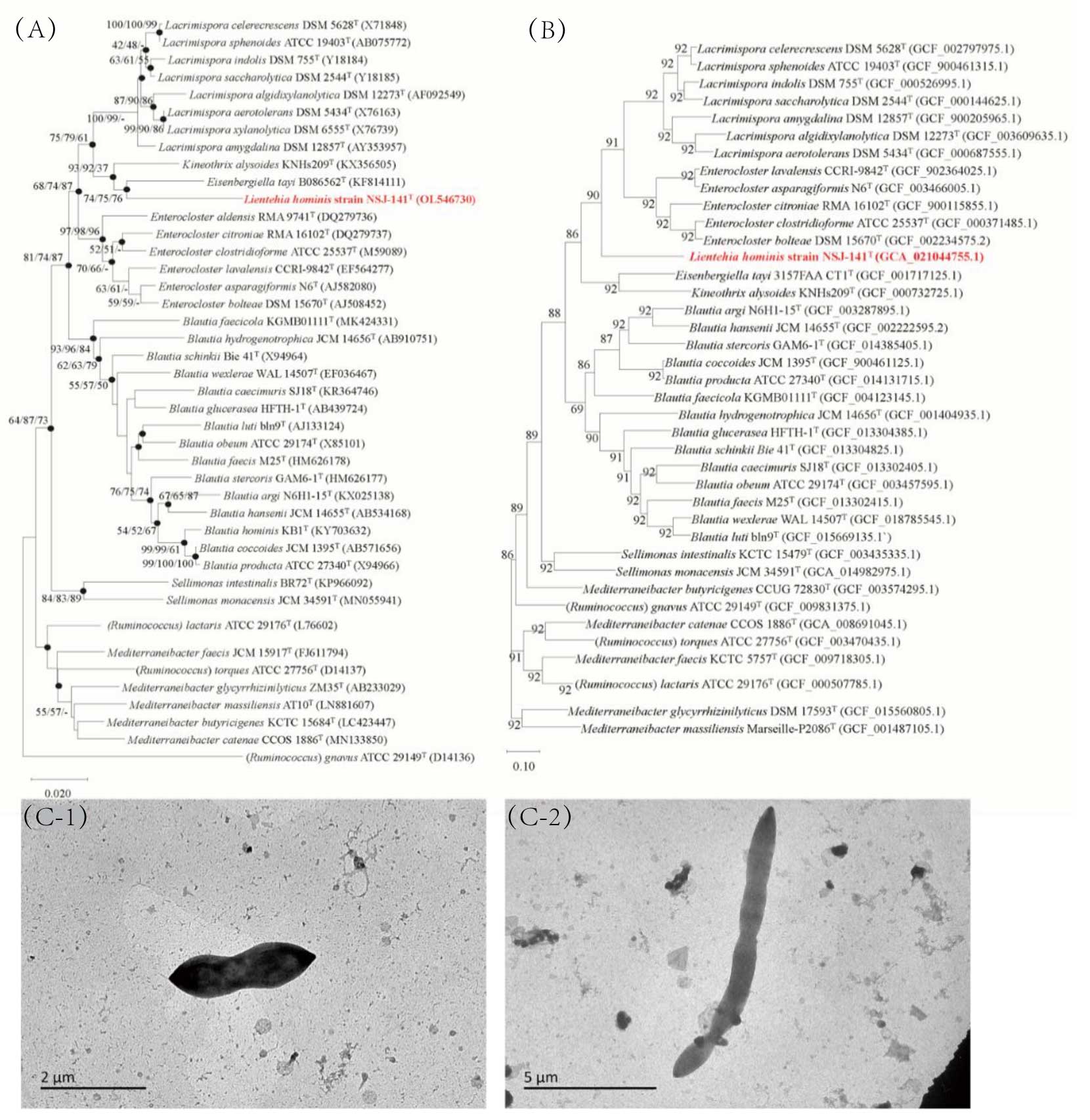


**Figure S5.** (A) Phylogenetic tree based on nearly complete 16S rRNA gene sequences showing the relationship between strain NSJ-141^T^ and its phylogenetic neighbours. This tree was constructed by using maximum-likelihood method with K2+G+I type with 1,000 bootstraps, phylogenetic trees based on the maximum-parsimony and the neighbour-joining methods with 1,000 bootstraps were also cunstructed, and bootstrap values (percentage of replicates) above the threshold of ≥50 % are shown for those nodes supported in at least one of the three methods; these bootstrap values are depicted in the order NJ/ML/MP. Filled circles indicate nodes reconstructed by all three methods. GenBank accession numbers are given in parentheses. Bar, 0.02 substitutions per nucleotide position; (B) Phylogenomic tree of strain NSJ-141^T^ and closely related strains based on 92 bacterial core gene sequences constructed using Up-to-date bacterial core genes (UBCG) of the genomes of related strains available on NCBI GenBank. GenBank accession numbers are shown in parentheses. Gene Support Index (GSI) values of 92 UBCGs are given at branching points; (C-1, C-2) Transmission electron micrograph of NSJ-141^T^ cells grown on Lach-GAM agar.


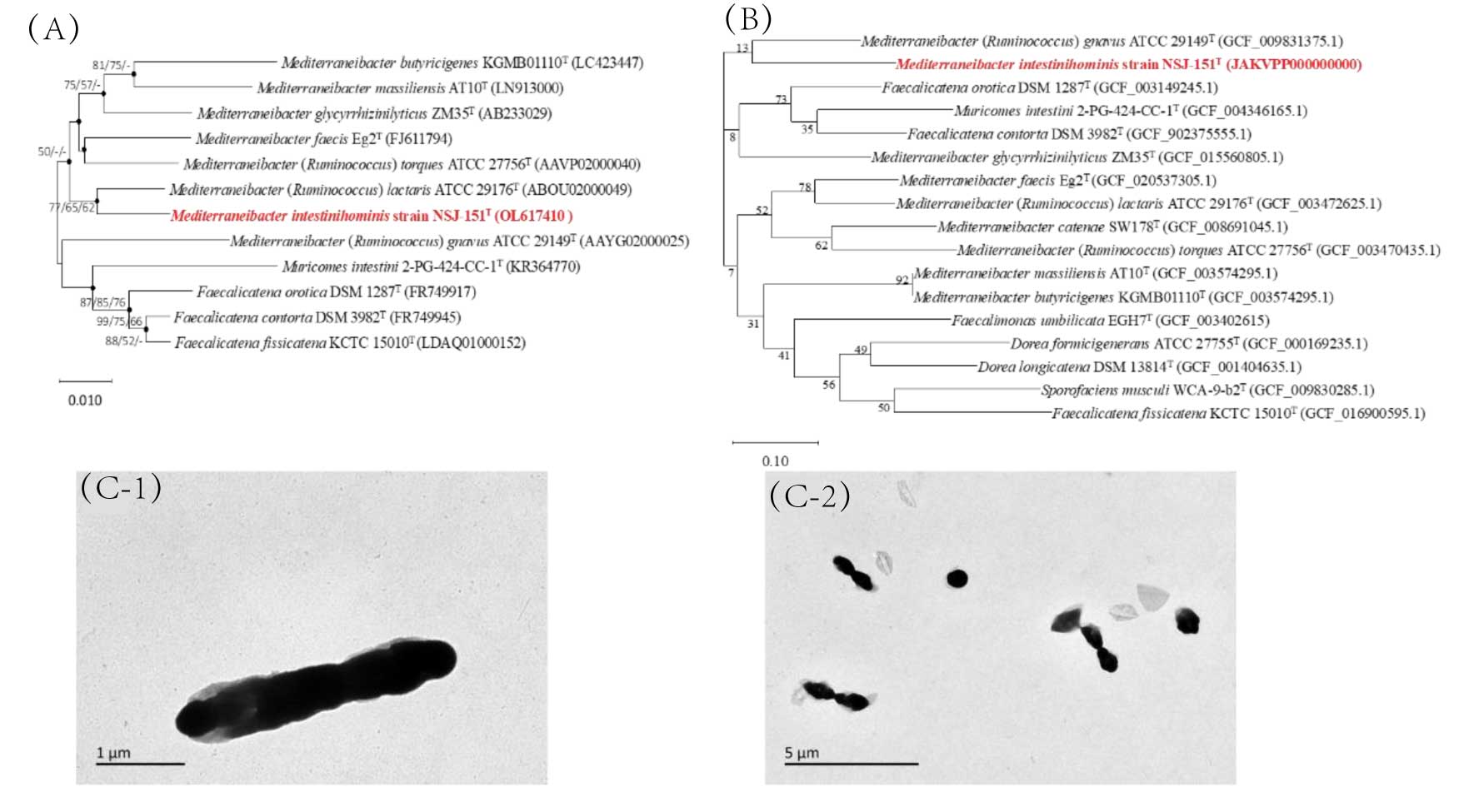


**Figure S6.** (A) Phylogenetic tree based on nearly complete 16S rRNA gene sequences showing the relationship between strain NSJ-151^T^ and its phylogenetic neighbours. This tree was constructed by using maximum-likelihood method with K2+G+I type with 1,000 bootstraps, phylogenetic trees based on the maximum-parsimony and the neighbour-joining methods with 1,000 bootstraps were also cunstructed, and bootstrap values (percentage of replicates) above the threshold of ≥50 % are shown for those nodes supported in at least one of the three methods; these bootstrap values are depicted in the order NJ/ML/MP. Filled circles indicate nodes reconstructed by all three methods. GenBank accession numbers are given in parentheses. Bar, 0.01 substitutions per nucleotide position; (B) Phylogenomic tree of strain NSJ-151^T^ and closely related strains based on 92 bacterial core gene sequences constructed using Up-to-date bacterial core genes (UBCG) of the genomes of related strains available on NCBI GenBank. GenBank accession numbers are shown in parentheses. Gene Support Index (GSI) values of 92 UBCGs are given at branching points; (C-1, C-2) Transmission electron micrograph of NSJ-151^T^ cells grown on Lach-GAM agar.

**
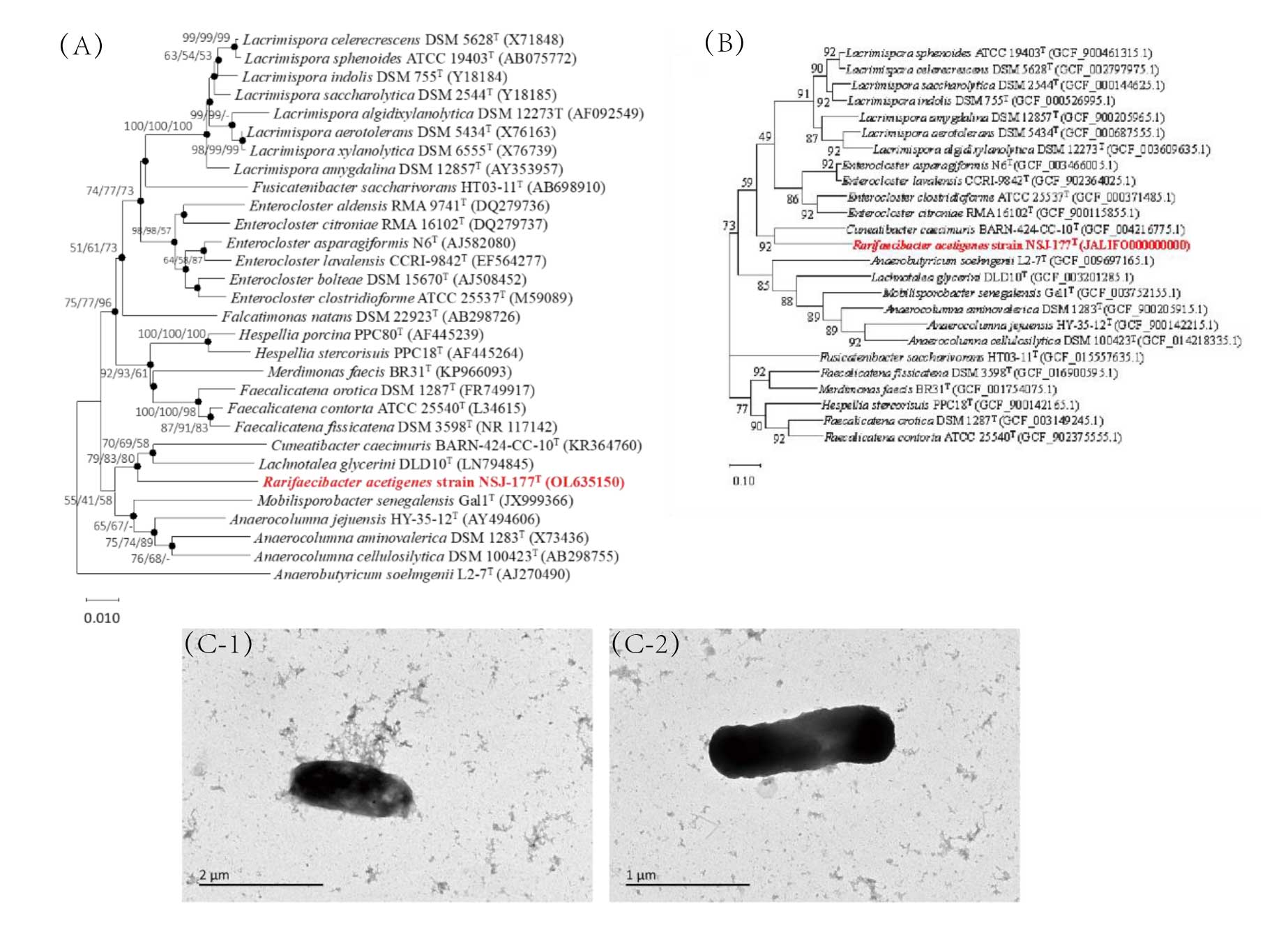
Figure S7.** (A) Phylogenetic tree based on nearly complete 16S rRNA gene sequences

showing the relationship between strain NSJ-177^T^ and its phylogenetic neighbours.

This tree was constructed by using maximum-likelihood method with K2+G+I type

with 1,000 bootstraps, phylogenetic trees based on the maximum-parsimony and the

neighbour-joining methods with 1,000 bootstraps were also cunstructed, and bootstrap

values (percentage of replicates) above the threshold of ≥50 % are shown for those

nodes supported in at least one of the three methods; these bootstrap values are

depicted in the order NJ/ML/MP. Filled circles indicate nodes reconstructed by all

three methods. GenBank accession numbers are given in parentheses. Bar, 0.01

substitutions per nucleotide position; (B) Phylogenomic tree of strain NSJ-177^T^ and

closely related strains based on 92 bacterial core gene sequences constructed using

Up-to-date bacterial core genes (UBCG) of the genomes of related strains available on

NCBI GenBank. GenBank accession numbers are shown in parentheses. Gene

Support Index (GSI) values of 92 UBCGs are given at branching points; (C-1, C-2)

Transmission electron micrograph of NSJ-177^T^ cells grown on Lach-GAM agar.


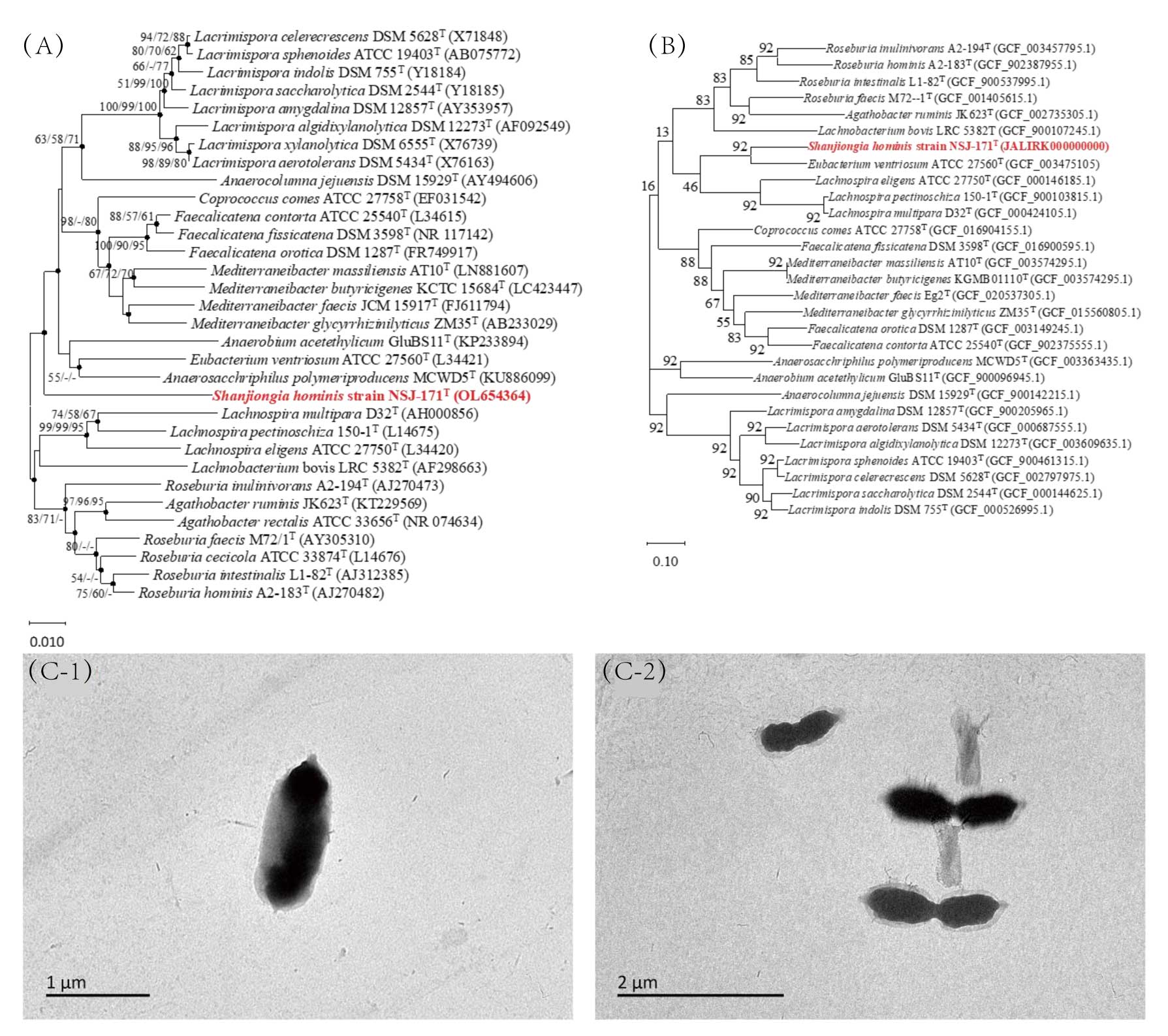


**Figure S8.** (A) Phylogenetic tree based on nearly complete 16S rRNA gene sequences showing the relationship between strain NSJ-171^T^ and its phylogenetic neighbours. This tree was constructed by using maximum-likelihood method with K2+G+I type with 1,000 bootstraps, phylogenetic trees based on the maximum-parsimony and the neighbour-joining methods with 1,000 bootstraps were also cunstructed, and bootstrap values (percentage of replicates) above the threshold of ≥50 % are shown for those nodes supported in at least one of the three methods; these bootstrap values are depicted in the order NJ/ML/MP. Filled circles indicate nodes reconstructed by all three methods. GenBank accession numbers are given in parentheses. Bar, 0.01 substitutions per nucleotide position; (B) Phylogenomic tree of strain NSJ-171^T^ and closely related strains based on 92 bacterial core gene sequences constructed using Up-to-date bacterial core genes (UBCG) of the genomes of related strains available on NCBI GenBank. GenBank accession numbers are shown in parentheses. Gene Support Index (GSI) values of 92 UBCGs are given at branching points; (C-1, C-2) Transmission electron micrograph of NSJ-171^T^ cells grown on Lach-GAM agar.
